# FAM71F1 binds to RAB2A and RAB2B and is essential for acrosome formation and male fertility in mice

**DOI:** 10.1242/dev.199644

**Published:** 2021-10-29

**Authors:** Akane Morohoshi, Haruhiko Miyata, Yuki Oyama, Seiya Oura, Taichi Noda, Masahito Ikawa

**Affiliations:** 1 Department of Experimental Genome Research, Research Institute for Microbial Diseases, Osaka University, Osaka 565-0871, Japan; 2 Graduate School of Medicine, Osaka University, Osaka 565-0871, Japan; 3 Graduate School of Pharmaceutical Sciences, Osaka University, Osaka 565-0871, Japan; 4 Division of Reproductive Biology, Institute of Resource Development and Analysis, Kumamoto University, Kumamoto 860-0811, Japan; 5 Priority Organization for Innovation and Excellence, Kumamoto University, Kumamoto 860-8555, Japan; 6 Laboratory of Reproductive Systems Biology, The Institute of Medical Science, The University of Tokyo, Tokyo 108-8639, Japan; 7 Center for Infectious Disease Education and Research (CiDER), Osaka University, Osaka 565-0871, Japan

**Keywords:** Acrosome, Spermatogenesis, Globozoospermia, Mouse

## Abstract

The acrosome is a cap-shaped, Golgi-derived membranous organelle that is located over the anterior of the sperm nucleus and highly conserved throughout evolution. Although morphological changes during acrosome biogenesis in spermatogenesis have been well described, the molecular mechanism underlying this process is still largely unknown. Family with sequence similarity 71, member F1 and F2 (FAM71F1 and FAM71F2) are testis-enriched proteins that contain a RAB2B-binding domain, a small GTPase involved in vesicle transport and membrane trafficking. Here, by generating mutant mice for each gene, we found that *Fam71f1* is essential for male fertility. In *Fam71f1*-mutant mice, the acrosome was abnormally expanded at the round spermatid stage, likely because of enhanced vesicle trafficking. Mass spectrometry analysis after immunoprecipitation indicated that, in testes, FAM71F1 binds not only RAB2B, but also RAB2A. Further study suggested that FAM71F1 binds to the GTP-bound active form of RAB2A/B, but not the inactive form. These results indicate that a complex of FAM71F1 and active RAB2A/B suppresses excessive vesicle trafficking during acrosome formation.

## Abstract (precis)

**Summary:** FAM71F1 interacts with RAB2A/B and regulates the formation of the acrosome in mice, the misregulation of which impacts male fertility.

## INTRODUCTION

Spermatozoa have a unique morphology specialized for fertilization, which is produced in the seminiferous tubules through a well-organized process called spermatogenesis (Jan et al., 2012). During mammalian spermatogenesis, diploid spermatogonia undergo mitosis and produce spermatocytes, which go through meiosis to become haploid round spermatids. These spermatids then undergo a morphological transformation, called spermiogenesis, which includes acrosome formation, nuclear elongation and tail formation, to form the mature spermatozoa.

The acrosome is a highly specialized membrane organelle that covers the anterior of the sperm nucleus and is often regarded as a ‘specially modified lysosome’ or ‘lysosome-related organelle’ because it contains numerous acid hydrolytic enzymes (Berruti and Paiardi, 2011; Khawar et al., 2019; Moreno and Alvarado, 2006). Spermatozoa need to undergo an exocytotic event, called the acrosome reaction, before fusing with eggs (Buffone et al., 2008). During the acrosome reaction, hydrolytic enzymes are released and acrosomal membrane proteins spread onto the sperm plasma membrane for successful fertilization. In mammals, the IZUMO1 membrane protein initially locates to the acrosomal membrane and is exposed on the surface of the sperm heads, where it binds to the oocyte through JUNO (IZUMO1 receptor), a GPI-anchored oolemma protein, to trigger the fusion process (Inoue et al., 2005; Satouh et al., 2012; Okabe, 2013).

Acrosome biogenesis during spermatogenesis has also been well described. Acrosome formation can be divided into four phases: the Golgi, cap, acrosome and maturation phases (Khawar et al., 2019). During the Golgi phase, numerous proacrosomal granules derived from the *trans*-Golgi apparatus fuse and form a single sizeable acrosomal vesicle associated with the anterior of the nuclear envelope. During the cap phase, the acrosome vesicle gradually increases in size and spreads over the surface of the nucleus to form a cap-like structure. As the nucleus elongates during the acrosome phase, the acrosome spreads to the dorsal edge, whereas the Golgi apparatus moves caudally. Finally, acrosome formation completes during the maturation phase.

Globozoospermia is a rare and severe disorder causing male infertility and is characterized by an absent or severely malformed acrosome (Dam et al., 2007a; Coutton et al., 2015). In humans, *DPY19L2* (Celse et al., 2021; Coutton et al., 2012; Harbuz et al., 2011; Koscinski et al., 2011; Méndez and Stillman, 2000) and *SPATA16* have been reported as causative genes of globozoospermia (Dam et al., 2007b; Karaca et al., 2014), and putative mutations have been reported in *PICK1* (Liu et al., 2010) and *ZPBP1* (*ZPBP*) (Yatsenko et al., 2012). In addition, analyses of mutant mice identified globozoospermia-related genes that function at various points in acrosome formation, such as the formation and trafficking of proacrosomal granules (e.g. *Gba2*, *Pick1* and *Gopc*) (Xiao et al., 2009; Yao et al., 2002; Yildiz et al., 2006), fusion of the proacrosomal granules to form the acrosome [e.g. *Vps54*, *Hrb* (*Agfg1*) and *AU040320*] (Guidi et al., 2018; Kang-Decker et al., 2001; Paiardi et al., 2011) and anchoring the acrosome to the nucleus [e.g. *Dpy19l2*, *Zpbp1* (*Zpbp*) and *Spaca1*] (Fujihara et al., 2012; Lin et al., 2007; Pierre et al., 2012). Although some factors have been identified, more factors need to be determined to understand fully acrosome formation/function and their clinical relevance.

In the present study, we focused on Golgi-associated RAB2B interactor (GARI; also known as FAM71F2) and its related proteins GARI like 1 (GARI-L1; also known as FAM71F1) to GARI like 5 (GARI-L5; also known as FAM71E1), because these proteins bind specifically to RAB2B (Fukuda et al., 2008), a small GTPase involved in vesicle transport and membrane trafficking (Stenmark, 2009). It has been reported that GARI and GARI-L proteins colocalize with RAB2B and function in the Golgi apparatus in mammalian cultured cells (Aizawa and Fukuda, 2015; Fukuda et al., 2008; Takahama et al., 2017). According to Mouse ENCODE transcriptome data, all GARI and GARI-L proteins are expressed strongly in the testis; however, their functions *in vivo* remain unknown. Among these, the expression of *GARI-L1* has been reported to be significantly downregulated in patients with male infertility (Malcher et al., 2013). Here, we generated knockout (KO) mice of *Gari-l1* (*Fam71f1*) and *Gari* (*Fam71f2*), the closest paralog of *Fam71f1*, and analyzed their functions in acrosome biogenesis.

## RESULTS

### FAM71F1 and FAM71F2 are evolutionarily conserved and testis-enriched proteins

**Fig. 1.**
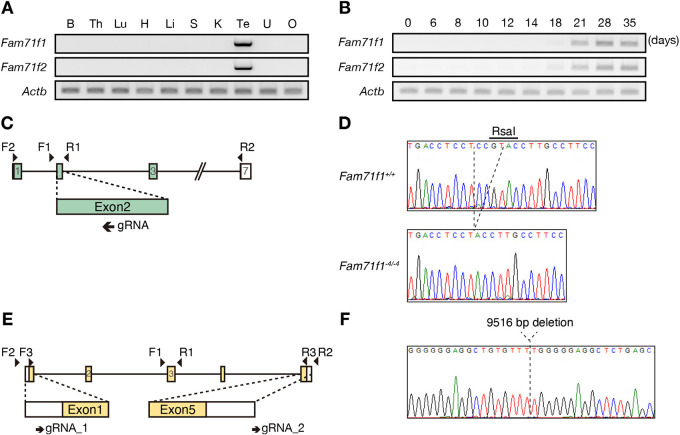
**Generation of *Fam71f1*- and *Fam71f2*-KO mice.** (A,B) The expression of mouse *Fam71f1* and *Fam71f2* was examined by RT-PCR using RNA isolated from various organs (A) and from testis at various postnatal days (B). Both *Fam71f1* and *Fam71f2* were enriched in testis (A). Strong signals of *Fam71f1* and *Fam71f2* were detected from postnatal day 21 (B). *Actb* was used as a loading control. (C) Targeting scheme for generating *Fam71f1*-KO mice with the CRISPR/Cas9 system. The gRNA was designed to target exon 2. Primers F1 and R1 were used for genotyping (Fig. S2A) and F2 and R2 were used for RT-PCR in A,B (Fig. S2B). (D) DNA sequence of *Fam71f1* in *Fam71f1^+/+^* and *Fam71f1^−4/−4^* mice. The RsaI recognition site (5′-GTAC-3′) was disrupted by the 4 bp deletion. (E) Targeting scheme for generating *Fam71f2*-KO mice with the CRISPR/Cas9 system. The gRNAs were designed to target exon 1 and exon 5. Primers F1, F2, R1 and R2 were used for genotyping (Fig. S2D) and F3 and R3 were used for RT-PCR in A,B (Fig. S2E). (F) DNA sequence of *Fam71f2*-mutant mice. The mutant allele had a 9516 bp deletion. B, brain; H, heart; K, kidney; Li, liver; Lu, lung; O, ovary; S, spleen; Te, testis; Th, thymus; U, uterus.

FAM71F1 and FAM71F2 are conserved in mammals, including mice and humans (Fig. S1A,B). To investigate the spatial expression of *Fam71f1* and *Fam71f2* in mice, we conducted multi-tissue PCR using cDNA obtained from adult mice. Both *Fam71f1* and *Fam71f2* were expressed strongly in the testis (Fig. 1A). In mice, the first wave of spermatogenesis completes within the first 35 days of postnatal development (Kluin et al., 1982). To determine the stage of spermatogenesis in which *Fam71f1* and *Fam71f2* start to be expressed, we conducted PCR using cDNA obtained from the postnatal testis. Both genes were expressed from day 21, around when spermiogenesis occurs (Fig. 1B). Furthermore, *Fam71f1* and *Fam71f2* expression dramatically increases at the mid-round spermatid stage (steps 4-6), according to published single-cell RNA-sequencing data in mice (Fig. S1C) (Hermann et al., 2018). These results suggest that *Fam71f1* and *Fam71f2* function when flagellum elongation and head morphogenesis occur during spermiogenesis.

### Generation of *Fam71f1*- and *Fam71f2*-knockout mice

To uncover the function of *Fam71f1* and *Fam71f2 in vivo*, we generated *Fam71f1* or *Fam71f2* single-KO mice. To generate *Fam71f1*-KO mice, we designed one guide RNA (gRNA) targeting exon 2 (Fig. 1C) and inserted the target sequence into a pX330 plasmid, which expresses gRNA and humanized CAS9 (Mashiko et al., 2013). We injected the plasmid into the pronuclei of fertilized oocytes and transplanted the developed two-cell embryos into the oviduct of pseudopregnant female mice, which generated a mutant mouse line with a 4 bp deletion. We confirmed the deletion by sequencing and RsaI digestion of the genomic PCR product (Fig. 1D; Fig. S2A). Indel mutations can lead to alternative splicing, but only one mRNA variant with an expected 4 bp deletion was detected with full-length RT-PCR (Fig. S2B). This 4 bp deletion caused a frame-shift mutation at S107Y and a subsequent premature stop codon (Fig. S2C).

For *Fam71f2*-KO mice, we designed two gRNAs targeting the 5′ untranslated region of exon 1 and the 3′ downstream region of exon 5 in the *Fam71f2* locus (Fig. 1E). We electroporated fertilized oocytes with the CAS9-gRNA ribonucleoproteins and transplanted developed two-cell embryos into the oviduct of pseudopregnant female mice. We confirmed the large deletion by genomic PCR (Fig. S2D), and subsequent sequence analysis revealed a 9516 bp deletion in the *Fam71f2* locus (Fig. 1F). The absence of *Fam71f2* mRNA in the *Fam71f2^−9516/−9516^* testis was confirmed with full-length RT-PCR (Fig. S2E).

### *Fam71f1* and *Fam71f2* are required for normal male fertility and sperm head formation

**Fig. 2.**
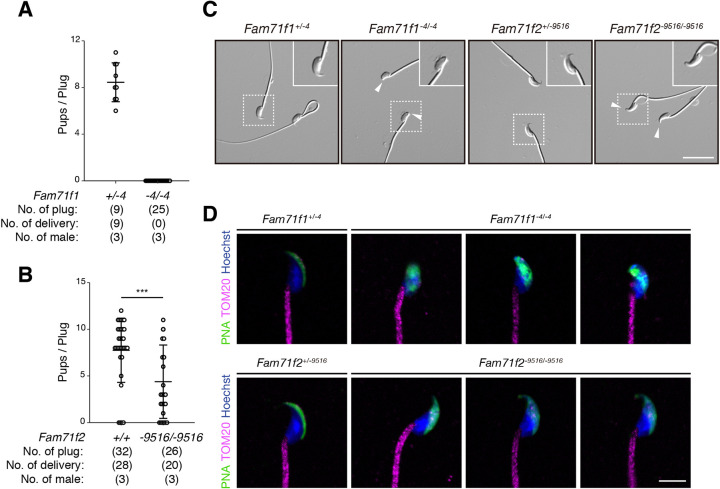
***Fam71f1* and *Fam71f2* are required for normal male fertility and sperm head formation.** (A) Fertility of *Fam71f1*-mutant males. *Fam71f1^+/−4^* or *Fam71f1^−4/−4^* males were mated with wild-type (WT) females and the number of pups born per plug was counted. Although 25 plugs were observed, *Fam71f1^−4/−4^* males did not sire any offspring. (B) Fertility of *Fam71f2*-mutant males. *Fam71f2^+/+^* or *Fam71f2^−9516/−9516^* males were mated with WT females and the number of pups born per plug was counted. The fertility of *Fam71f2^−9516/−9516^* males was significantly decreased compared with WT. (C) Observation of spermatozoa. Spermatozoa from *Fam71f1*- and *Fam71f2*-mutant mice lacked hook-like structures at the apical regions of sperm heads (arrowheads). Higher magnification images are shown in the top-right corners. (D) Immunofluorescence staining of the acrosome and mitochondria. Both *Fam71f1*- and *Fam71f2*-mutant spermatozoa showed abnormal morphology of the acrosome, whereas the mitochondrial sheaths were normal. Nuclei are stained blue (Hoechst), acrosomes green (lectin-PNA) and mitochondria magenta (TOM20). Data are mean±s.d.; ***P<0.001 (unpaired Student's *t*-test). Scale bars: 20 µm in C; 5 µm in D.

To analyze *Fam71f1^−4/−4^* and *Fam71f2^−9516/−9516^* mouse fertility, individual males were caged with wild-type females for 2 months. We found that *Fam71f1^−4/−4^* males failed to sire any pups despite successful matings with vaginal plugs [average litter size: 8.4±1.6 (*Fam71f1^+/−4^* males); 0 (*Fam71f1^−4/−4^* males)] (Fig. 2A). *Fam71f2^−9516/−9516^* male fertility was significantly impaired compared with that of the controls [average litter size: 7.2±3.4 (*Fam71f2^+/+^*); 4.4±3.9 (*Fam71f2^−9516/−9516^*)] (Fig. 2B).

To investigate why the fertility of *Fam71f1^−4/−4^* and *Fam71f2^−9516/−9516^* male mice was impaired, we observed the morphology of testis and spermatozoa obtained from the cauda epididymis. The testis appearance, size and weight in both *Fam71f1^−4/−4^* and *Fam71f2^−9516/−9516^* males were comparable to those of heterozygous mice (Fig. S3A-D); however, spermatozoa from these homozygous mutant mice showed abnormal head shapes (Fig. 2C) (number of spermatozoa with abnormal head morphology: 164/164 spermatozoa examined for *Fam71f1^−4/−4^* mice and 150/150 spermatozoa examined for *Fam71f2^−9516/−9516^* mice; n=3 males for each group). Hook-like structures at the apical sperm heads were abnormal in both types of mutant spermatozoa, although the morphological abnormality was milder in *Fam71f2*-mutant spermatozoa.

In humans, globozoospermia is characterized by malformation or loss of the acrosome (Dam et al., 2007a) and, in some cases, an abnormal mitochondrial sheath that wraps around the sperm head is observed (Battaglia et al., 1997). Therefore, we observed acrosomes and mitochondria using PNA, which stains the acrosome, and anti-TOM20 antibodies, which stain the mitochondria (Fig. 2D; Fig. S3E). Although control spermatozoa from these heterozygous mice exhibited a crescent moon-shaped acrosome, both *Fam71f1*-mutant and *Fam71f2*-mutant spermatozoa showed an abnormal acrosome morphology. In *Fam71f1^−4/−4^* mice, the acrosome was abnormally spread over the entire sperm head, whereas, in *Fam71f2^−9516/−9516^* mice, the acrosome exhibited a slightly expanded crescent shape. In contrast, the morphology of the mitochondria was normal in both mutant spermatozoa. Furthermore, we performed immunofluorescence for IZUMO1, which is localized in the inner and outer acrosomal membrane before the acrosome reaction. Consistent with abnormal acrosome morphology, IZUMO1 localization was impaired in both *Fam71f1*-mutant and *Fam71f2*-mutant spermatozoa (Fig. S3F,G). These results indicate that both *Fam71f1* and *Fam71f2* are required for accurate acrosome formation and normal male fertility.

### *Fam71f1* but not *Fam71f2* is required for the acrosome reaction

**Fig. 3.**
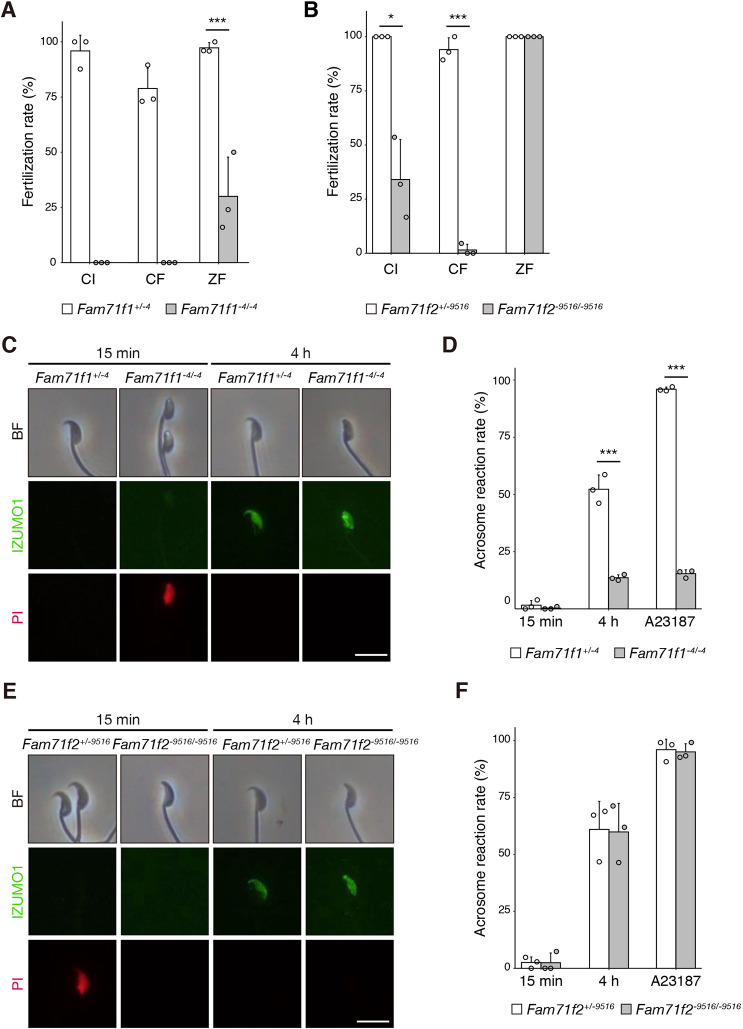
***Fam71f1* but not *Fam71f2* is required for the acrosome reaction.** (A) IVF assay of *Fam71f1*-mutant males. *Fam71f1*-mutant spermatozoa were unable to fertilize CI or CF eggs. In contrast, the fertilization rate (percentage of two pronuclei observed 8 h after insemination) was partially rescued with ZF eggs. (B) IVF assay of *Fam71f2*-mutant males. The fertilization rates with CI and CF eggs were significantly lower in *Fam71f2^−9516/−9516^* mice compared with *Fam71f2^+/−9516^* mice. In contrast, the fertilization rate was rescued with ZF eggs. (C) Immunofluorescence staining for IZUMO1 (green) of live *Fam71f1*-mutant spermatozoa. After the acrosome reaction (4 h incubation), IZUMO1 spread to the equatorial segment in the control. However, in *Fam71f1*-mutant spermatozoa, IZUMO1 localization was abnormal, although it was exposed on the surface of the acrosome. PI (red), which stains nuclei of dead spermatozoa, was used to distinguish live cells from dead cells. (D) The acrosome reaction rates of *Fam71f1*-mutant spermatozoa at 15 min and 4 h after incubation in capacitation medium. After 4 h incubation, the Ca^2+^ ionophore A23187 was added to induce the acrosome reaction. Live spermatozoa with IZUMO1 signal were considered acrosome reacted. After 4 h of incubation and A23187 addition, the acrosome reaction rates were significantly lower in *Fam71f1^−4/−4^* mice compared with *Fam71f1^+/−4^* mice. (E) Immunofluorescence staining for IZUMO1 of live *Fam71f2*-mutant spermatozoa. After the acrosome reaction (4 h incubation), IZUMO1 spread to the equatorial segment in both the control and *Fam71f2*-mutant spermatozoa. PI was used to distinguish live cells from dead cells. (F) The acrosome reaction rates of *Fam71f2*-mutant spermatozoa at 15 min and 4 h after incubation in capacitation medium. After 4 h incubation, A23187 was added to induce the acrosome reaction. Live spermatozoa with IZUMO1 signal were considered acrosome reacted. There were no significant differences in acrosome reaction rates between *Fam71f2^+/−9516^* and *Fam71f2^−9516/−9516^* mice. Error bars represent s.d. *n*=3 males in each group; *P<0.05, ***P<0.001 (unpaired Student's *t*-test). Scale bars: 10 µm in C,E.

When we performed *in vitro* fertilization (IVF), *Fam71f1*-mutant spermatozoa were unable to fertilize cumulus-intact (CI) or cumulus-free (CF) zona pellucida (ZP)-intact oocytes; however, 30.0% of *Fam71f1*-mutant spermatozoa fused with ZP-free oocytes (ZP free; ZF) (Fig. 3A). For *Fam71f2^−9516/−9516^* mice, fertilization rates were significantly decreased with both CI and CF oocytes, but no difference was observed with ZF oocytes (Fig. 3B). Furthermore, we did not observe any spermatozoa in the perivitelline space in either mutant mouse type, which was seen with the mutant spermatozoa lacking any known oocyte-fusing factors (Fujihara et al., 2020; Noda et al., 2020). These results indicate that *Fam71f1*-mutant and *Fam71f2*-mutant spermatozoa have problems penetrating the ZP.

Given that decreased sperm motility leads to impaired ZP penetration (Miyata et al., 2015), we examined sperm motility using a computer-assisted sperm analyzer (CASA). The percentage of motile spermatozoa was slightly decreased in *Fam71f1* mutants after incubation in a capacitating medium (Fig. S4A). In addition, there was a slight decrease in velocity parameters examined, such as average path velocity (VAP), straight-line velocity (VSL) and curvilinear velocity (VCL) (Fig. S4B). However, all velocity parameters of *Fam71f1*-mutant spermatozoa (B6D2 mixed background) were comparable to those of C57BL/6 wild-type spermatozoa (Miyata et al., 2015). For *Fam71f2*-mutant mice, no differences were observed in the percentage of motile spermatozoa or any of the velocity parameters (Fig. S4C,D). These results suggest that sperm motility alone cannot explain the impaired ZP penetration in either *Fam71f1*-mutant or *Fam71f2*-mutant mice.

We also analyzed the acrosome reaction necessary for penetrating the ZP (Wassarman, 1999) by conducting live-sperm immunofluorescence for IZUMO1. As mentioned above, IZUMO1 is located in the inner and outer acrosomal membrane before the acrosome reaction and does not react with anti-IZUMO1 antibodies with the live-staining method. In contrast, IZUMO1 is exposed on the surface after the acrosome reaction and reacts with anti-IZUMO1 antibodies (Satouh et al., 2012). After a 15 min incubation in a capacitation medium, no IZUMO1 signal was detected in most of the spermatozoa in either *Fam71f1*-mutant or *Fam71f2*-mutant mice (Fig. 3C-F). After a 4 h incubation, the rates of IZUMO1-positive spermatozoa were comparable between control and *Fam71f2^−9516/−9516^* mice (Fig. 3E,F); however, the rate was significantly lower in *Fam71f1^−4/−4^* mice compared with control mice (Fig. 3C,D), indicating that the acrosome reaction was impaired in *Fam71f1*-mutant but not *Fam71f2*-mutant mice. The acrosome reaction was not induced even with the Ca^2+^ ionophore, A23187, in *Fam71f1*-mutant spermatozoa (Fig. 3D). Furthermore, impaired acrosome reactions in *Fam71f1^−4/−4^* mice were confirmed by using *Acr*-*EGFP* transgenic (Tg) mice the spermatozoa of which lose the EGFP signal after the acrosome reaction (Fig. S4E) (Nakanishi et al., 1999). These results suggest that the ZP penetration failure is due to an impaired acrosome reaction in addition to abnormal sperm head morphology in *Fam71f1^−4/−4^* mice. In contrast, abnormal sperm head morphology might be the cause of ZP penetration failure in *Fam71f2^−9516/−9516^* mice.

### *Fam71f1*-mutant spermatozoa exhibit a swollen acrosome

**Fig. 4.**
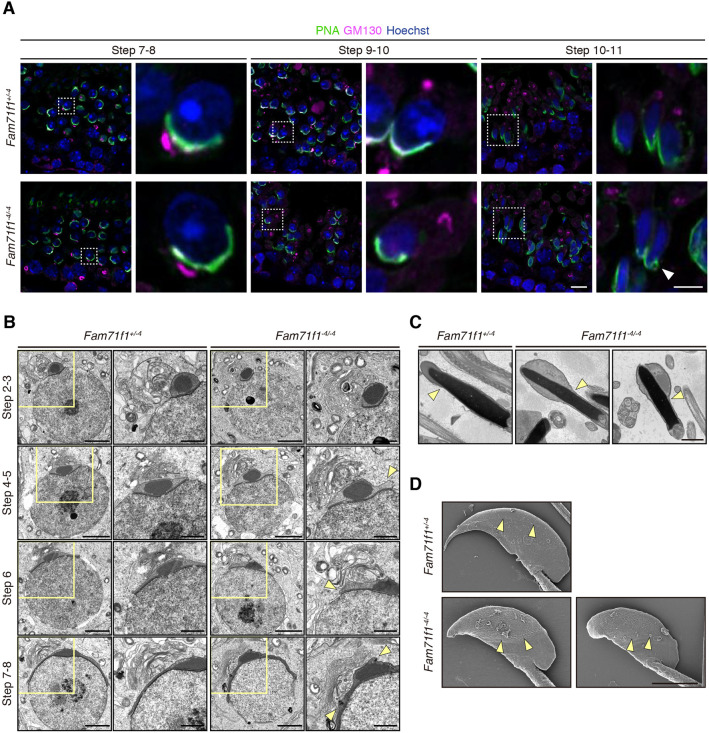
***Fam71f1*-mutant spermatozoa exhibit a swollen acrosome.** (A) Immunofluorescence staining of the acrosome and Golgi apparatus during spermatogenesis using testis sections. In round spermatids around steps 7 to 8, the morphology of the acrosome was comparable to that of the control; by contrast, in the elongated spermatids around steps 10 to 11, abnormal morphology of the acrosome was observed (arrowhead). The morphology and location of the Golgi apparatus were comparable to those of the controls. Higher magnification images of the boxed areas are shown to the right. Nuclei are stained blue (Hoechst), acrosomes green (lectin-PNA) and Golgi apparatus magenta (GM130). (B) TEM observation of the acrosome formation. Abnormal swelling of the acrosome was observed from around steps 4 to 5 in round spermatids in *Fam71f1^−4/−4^* mice (arrowheads). The morphology of the Golgi apparatus was comparable to that of the control. Higher magnification images of the boxed areas are shown to the right. (C) TEM observation of mature spermatozoa in the cauda epididymis. The acrosome was swollen and boundaries between the acrosomal cap and equatorial segment (arrowheads) were expanded in *Fam71f1*-mutant spermatozoa. (D) SEM observation of mature spermatozoa extracted from the cauda epididymis. Boundaries between the acrosomal cap and equatorial segment (arrowheads) were expanded in *Fam71f1*-mutant spermatozoa. Scale bars: 10 μm in A; 5 μm in A (right); 3 μm in D; 2 μm in B; 1 μm in B (right),C.

Given that FAM71F1 had a more severe phenotype in acrosome formation, acrosome reaction and male fertility compared with FAM71F2, we focused on FAM71F1 for further analysis. We first observed acrosome formation during spermatogenesis by immunofluorescence. The acrosome appeared normal in *Fam71f1^−4/−4^* mice until step 9; however, a swollen acrosome was observed from around step 10 to step 11 (Fig. 4A; Fig. S5A). In contrast, we found that the position and morphology of the Golgi apparatus appeared normal in *Fam71f1^−4/−4^* mice throughout all steps of spermatogenesis. The Golgi apparatus was located close to the acrosome in steps 2-8 and migrated to the caudal region after step 9 (Fig. 4A; Fig. S5A).

We next observed the early stages of acrosome formation in more detail by transmission electron microscopy (TEM). Abnormal swelling of the acrosome appeared around step 5 in *Fam71f1^−4/−4^* testis (Fig. 4B; Fig. S5B). A swollen acrosome remained evident in the cauda epididymal spermatozoa by TEM and scanning electron microscopy (SEM) observations (Fig. 4C,D). By contrast, no differences were observed in the Golgi apparatus (Fig. 4B), consistent with immunofluorescence staining (Fig. 4A; Fig. S5A).

### Infertility of *Fam71f1^−4/−4^* males is rescued with a transgene

Given the unavailability of effective antibodies against FAM71F1, we generated Tg mice expressing PA-tagged *Fam71f1* under the testicular germ cell-specific *Clgn* promoter (Ikawa et al., 2001; Watanabe et al., 1995) (Fig. S6A,B). *Fam71f1^−4/−4^* males carrying a *Fam71f1-PA* transgene (*−4/−4*; Tg) were caged with wild-type females for 2 months. We found that the infertility of *Fam71f1^−4/−4^* males was rescued with this transgene (Fig. S6C). Furthermore, FAM71F1-PA was detected in the testis but not in spermatozoa by immunoblot analysis (Fig. S6D), suggesting that FAM71F1 functions during spermatogenesis but not in mature spermatozoa.

### Relationship between *Fam71f1* and other globozoospermia-related proteins

To investigate the cause of acrosome malformation in the *Fam71f1* mutants, we examined the proteins known to be involved in acrosome formation. Immunoblot analysis of *Fam71f1^+/−4^* and *Fam71f1^−4/−4^* testis lysates revealed that the amount of GOPC (Yao et al., 2002) or ZPBP1 (Lin et al., 2007) had not changed. In contrast, some bands of SPACA1 (Fujihara et al., 2012) disappeared in the *Fam71f1^−4/−4^* testis (Fig. S7A). Further analysis using lysates collected from the spermatozoa in the cauda epididymis without incubation in a capacitation medium showed more-apparent band shifts in *Fam71f1^−4/−4^* mice (Fig. S7B). Given that SPACA1 has been reported to be *N*-linked glycosylated (Fujihara et al., 2012), we treated testis and sperm lysates with peptide-*N*-glycosidase F (PNGase F). The band patterns became comparable between *Fam71f1^+/−4^* and *Fam71f1^−4/−4^* testis lysates but not in sperm lysates after PNGase F treatment (Fig. S7C), suggesting that *N*-linked glycosylation of SPACA1 was impaired in the *Fam71f1^−4/−4^* testis. In contrast, treatment of testis and sperm lysates with *O*-glycosidase did not affect the band patterns (Fig. S7D). There were still multiple bands of SPACA1 after PNGase F or *O*-glycosidase treatment, which might be because of alternative splicing or other protein modification of SPACA1. To investigate the possibility that the change in the band size in *Fam71f1^−4/−4^* testis affects SPACA1 localization, we performed immunofluorescence for SPACA1 in the testes. We found that the localization of SPACA1 was comparable between *Fam71f1^+/−4^* and *Fam71f1^−4/−4^* testis (Fig. S7E). Furthermore, we found no interaction between FAM71F1 and SPACA1 in the testis by immunoprecipitation using the Tg line (Fig. S7F). These results suggest that FAM71F1 does not function directly with SPACA1. Rather, impaired SPACA1 processing could be a secondary effect of abnormal acrosome formation.

### FAM71F1 is localized to the Golgi apparatus and interacts with RAB2A and RAB2B

**Fig. 5.**
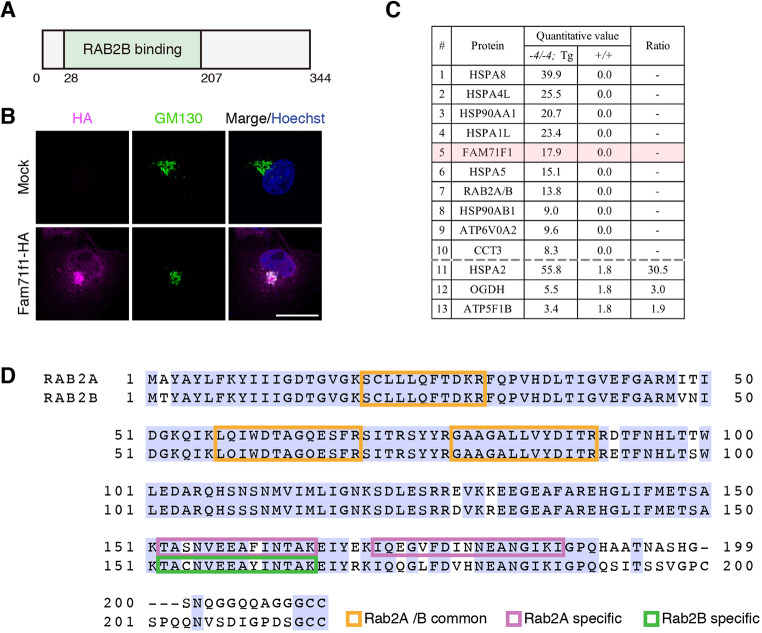
**FAM71F1 is localized to the Golgi apparatus and interacts with RAB2A and RAB2B.** (A) Domain structure of mouse FAM71F1. FAM71F1 contains a RAB2B-binding domain (green). (B) Immunofluorescence of FAM71F1 and Golgi apparatus using COS-7 cells. HA-tagged mouse *Fam71f1* was transiently expressed. FAM71F1-HA colocalized with GM130-positive Golgi apparatus. Nuclei are stained blue (Hoechst), Golgi apparatus green (GM130) and FAM71F1-HA magenta (HA). (C) The FAM71F1 proteome identified by immunoprecipitation-mass spectrometry analysis. Testis lysates from wild-type (*+/+*) or *Fam71f1-PA* Tg (*−4/−4*; Tg) mice were immunoprecipitated using anti-PA antibodies and associated proteins were identified by mass spectrometry. Proteins either identified only in PA-tagged *Fam71f1* Tg mice (1-10) or proteins with a high ratio in the Tg immunoprecipitation are listed (11-13). The remaining mass spectrometry results are shown in Table S1. Ratio=(*−4/−4*; Tg)/(+/+) (quantitative value). (D) Amino acid sequence of RAB2A and RAB2B. Peptide sequences of RAB2A and RAB2B that were identified by mass spectrometry are indicated by colored boxes; common sequences in RAB2A and RAB2B are in orange; specific sequences in RAB2A in magenta; and a specific sequence in RAB2B in green. Light-blue shading indicates amino acid residues common to both RAB2A and RAB2B. Scale bar: 20 μm in B.

In a previous study that analyzed Rab effectors comprehensively, FAM71F1 was identified as GARI-L1, which binds specifically to RAB2B. The N terminus (28-207 a.a.) of FAM71F1 is proposed to be the Rab-binding domain specific for RAB2B (Fukuda et al., 2008) (Fig. 5A). No other functional domains, including transmembrane domains (Phobius prediction) (Käll et al., 2007), were found in FAM71F1. Other GARI-L proteins and RAB2B have been shown to have several functions in the Golgi apparatus, such as in the maintenance of morphology and cytosolic DNA-induced IFN response (Aizawa and Fukuda, 2015; Takahama et al., 2017). Therefore, we examined the localization of FAM71F1 in cultured cells by immunofluorescence. When we expressed HA-tagged mouse FAM71F1 in COS-7 cells, FAM71F1 was colocalized with the GM130-positive Golgi apparatus (Fig. 5B). Next, we immunoprecipitated PA-tagged FAM71F1 using the mouse Tg line and conducted mass spectrometry analysis to investigate the interaction between FAM71F1 and RAB2B in the testis. Mass spectrometry analysis detected RAB2B and additional proteins, such as heat-shock proteins, that interact with FAM71F1-PA (Fig. 5C; Table S1). In addition to RAB2B, RAB2A-specific peptide sequences were also found in this analysis (Fig. 5D).

**Fig. 6.**
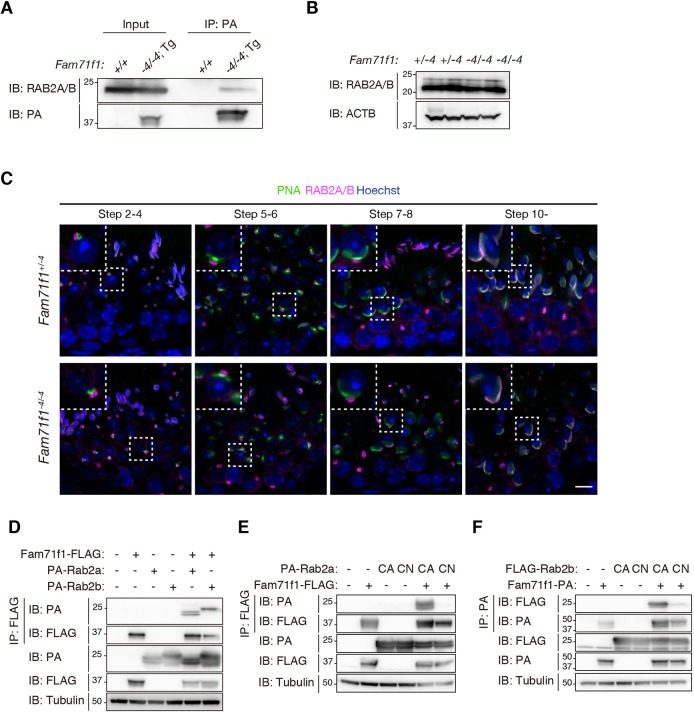
**FAM71F1 interacts with the constitutively active form of RAB2A/B.** (A) Immunoprecipitation of FAM71F1-PA using anti-PA antibodies. Testis lysates of wild-type (*+/+*) or *Fam71f1-PA* Tg (*−4/−4*; Tg) mice were used. FAM71F1-PA interacts with RAB2A/B. (B) Immunoblotting analysis for RAB2A/B using testis lysates. The amounts of RAB2A/B were comparable between *Fam71f1^+/−4^* and *Fam71f1^−4/−4^* mice. ACTB was used as a loading control. (C) Immunofluorescence staining of RAB2A/B and the acrosome during spermatogenesis using testis sections. RAB2A/B was localized in the Golgi apparatus until steps 7 to 8 and in the acrosome around step 10. No differences were found in the RAB2A/B localization between *Fam71f1^+/−4^* and *Fam71f1^−4/−4^* mice. Higher magnification images of the boxed areas are shown in the top-left. Nuclei are stained blue (Hoechst), acrosomes green (lectin-PNA) and RAB2A/B magenta. (D) Immunoprecipitation of FAM71F1-FLAG using anti-FLAG antibodies. *Fam71f1-FLAG* was transiently expressed with *PA-Rab2a* or *PA-Rab2b* in HEK293T cells. FAM71F1-FLAG interacts with both PA-RAB2A and PA-RAB2B. (E) Immunoprecipitation of FAM71F1-FLAG using anti-FLAG antibodies. *PA-Rab2a* (CA or CN) and *Fam71f1-FLAG* were transiently expressed in HEK293T cells. FAM71F1 interacts with RAB2A (CA). (F) Immunoprecipitation of FAM71F1-PA using anti-PA antibodies. *FLAG-Rab2b* (CA or CN) and *Fam71f1-PA* were transiently expressed in HEK293T cells. FAM71F1 interacts with RAB2B (CA). Tubulin was used as a loading control in D-F. Scale bar: 10 μm in C. CA, constitutively active (Q65L); CN, constitutively negative (S20N).

Using pan-RAB2 antibodies that detect both RAB2A and RAB2B (Fig. S8A), we confirmed using immunoprecipitation that FAM71F1 interacts with RAB2A/B in the testis (Fig. 6A). No differences were found in the amount of RAB2A/B between *Fam71f1^+/−4^* and *Fam71f1^−4/−4^* testes with immunoblot analysis (Fig. 6B). We then analyzed the localization of RAB2A/B in the testis with immunofluorescence. RAB2A/B was localized in the Golgi apparatus in the round spermatids and in the acrosome in the elongating spermatids (Fig. 6C; Fig. S8B). No impairment was observed in the localization of RAB2A/B in *Fam71f1^−4/−4^* testis (Fig. 6C). Considering that the acrosomal abnormality was found around step 5 (Fig. 4B), RAB2A/B in the Golgi apparatus might play roles in acrosome formation.

Using immunoprecipitation by expressing FLAG-tagged mouse FAM71F1 and PA-tagged mouse RAB2A or RAB2B in HEK293T cells, we confirmed that FAM71F1 interacts with both RAB2A and RAB2B (Fig. 6D). Other GARI proteins are known to interact with the GTP-bound active form of RAB2B (Aizawa and Fukuda, 2015; Fukuda et al., 2008; Takahama et al., 2017). Given that constitutively active (CA) and constitutively negative (CN) RAB2A/B protein states can be mimicked by point mutations (Q65L for CA and S20N for CN, respectively) (Aizawa and Fukuda, 2015), we co-expressed mouse FAM71F1 and CA or CN forms of mouse RAB2A or RAB2B in HEK293T cells. With immunoprecipitation and immunoblot analysis, we found that FAM71F1 interacts with the CA form of RAB2A/B but not with the CN forms (Fig. 6E,F).

## DISCUSSION

By generating mutant mice for *Fam71f1* and *Fam71f2* with the CRISPR/Cas9 system, we reveal that both genes are crucial for acrosome formation and male fertility. Their phenotypes were slightly different, with *Fam71f1^−4/−4^* mice showing more severe phenotypes resulting in male sterility. These results suggest that FAM71F1 and FAM71F2 might play similar roles in acrosome formation but that FAM71F1 is indispensable. We also reveal that *Fam71f1*-mutant spermatozoa exhibit impaired acrosome reactions with abnormal localization of IZUMO1. Impaired acrosome reactions have also been reported in other KO mouse lines that exhibit abnormal acrosome morphology (Wang et al., 2014).

In *Fam71f1*-mutant testis and spermatozoa, abnormal SPACA1 bands were observed by immunoblot analysis (Fig. S7B). Given that we could not confirm a physical interaction between FAM71F1 and SPACA1 in the testis (Fig. S7F), abnormal SPACA1 processing might result from a secondary effect of abnormal acrosome formation. Although apparent differences in molecular weights of SPACA1 were observed in spermatozoa between control and *Fam71f1^−4/−4^* mice, slight abnormalities were already detected in the testis, caused by abnormal *N*-linked glycosylation (Fig. S7C). *Fam71f1* depletion might cause a functional abnormality of the Golgi apparatus, in which protein glycosylation occurs (Stanley, 2011). SPACA1 is further processed when spermatozoa migrate in the epididymis, which is impaired in *Fam71f1^−4/−4^* mice (Fig. S7B). Given that FAM71F1 was not detected in the mature spermatozoa (Fig. S6D), this impairment in spermatozoa collected from the cauda epididymis might also be a secondary effect of abnormal acrosome formation. It is also possible that impaired *N*-linked glycosylation in the testis affects SPACA1 processing in the epididymis.

We found that *Fam71f1* mutant spermatozoa show abnormal swelling of the acrosome. Furthermore, immunoprecipitation analysis revealed that FAM71F1 interacts with not only RAB2B, but also RAB2A. Given that FAM71F1 interacts with the active forms but not the negative forms of RAB2A and RAB2B, FAM71F1 is probably involved in acrosome formation by regulating the function of activated RAB2A and RAB2B. Several studies indicate that RAB2 proteins regulate vesicle size (Kajiho et al., 2016; Lörincz et al., 2017). In human breast cancer cell lines, the expression of constitutively active RAB2A increased the size of RAB7-positive vesicles (Kajiho et al., 2016). In *Drosophila*, there is only one RAB2, and the expression of constitutively active RAB2 results in increased autolysosome size (Lörincz et al., 2017). This study suggests that active RAB2 is transported in Golgi-derived vesicles to fuse with RAB7-positive vesicles, such as autophagosomes and late endosomes. Considering that the acrosome is a RAB7-positive vesicle that derives from the Golgi apparatus (Ramalho-Santos and Moreno, 2001; Ramalho-Santos et al., 2001) and that RAB2A/B was localized in the Golgi apparatus when the abnormal acrosome formation was found (Fig. 4B; Fig. S8B), GTP-bound active RAB2A/B might be transported in Golgi-derived vesicles to regulate their fusion with the acrosome vesicle. Given that FAM71F1 is localized in the Golgi apparatus when it is expressed in COS-7 cells (Fig. 5B), it might bind to active RAB2A/B in the Golgi apparatus and negatively regulate these two proteins to form appropriately sized acrosomes.

In this study, we found that *Fam71f1* is essential for acrosome formation and male fertility. *Fam71f1* is conserved in humans, and its dysfunction might lead to globozoospermia. Further analysis of the function of FAM71F1 and its relationship with membrane trafficking-related proteins, such as RAB2A and RAB2B, will help to understand the mechanism that regulates acrosome formation and identify causative genes of globozoospermia.

## MATERIALS AND METHODS

### Animals

All mice used in this study were purchased from CLEA Japan or Japan SLC. All mice were maintained under specific-pathogen-free conditions in a temperature-controlled environment, under a 12-h light/12-h dark cycle and with *ad libitum* feeding. All animal experiments were conducted in accordance with the guidelines established by the Research Institute for Microbial Diseases, Osaka University (Japan), and were approved by the Animal Care and Use Committee of the Research Institute for Microbial Diseases, Osaka University (#Biken-AP-H30-01). All gene-modified mice generated in this study will be made available through either the RIKEN BioResource Research Center (Ibaraki, Japan) or the Center for Animal Resources and Development (CARD), Kumamoto University (Japan).

### Comparison of amino acid sequences

Amino acid sequences of each protein were obtained from the NCBI Entrez Protein database. The accession numbers of each amino acid sequence used were as follows: mouse FAM71F1 (NP_001276592.1), human FAM71F1 (NP_001269717.1), mouse FAM71F2 (NP_001094956.1), human FAM71F2 (NP_001012457.3), mouse RAB2A (NP_067493.1) and mouse RAB2B (NP_766189.1). Multiple sequence alignment was performed using Clustal Omega (https://www.ebi.ac.uk/Tools/msa/clustalo/) (Sievers et al., 2011).

### Isolation of RNA and RT-PCR

RNA was isolated and purified from multiple adult tissues of C57BL/6N mice at different ages using TRIzol (15596018, Thermo Fisher Scientific) according to the manufacturer's protocol. Reverse transcription was performed using purified RNA and the SuperScript III first-strand synthesis system (18080051, Thermo Fisher Scientific). PCR was carried out using KOD Fx Neo (KFX-201, TOYOBO). The primers for each gene are listed in Table S2.

### *In silico* expression analysis in spermatogenic cells

mRNA expression of *Fam71f1* and *Fam71f2* in testicular germ cells was analyzed by the Loupe Cell Browser 3.3.1 (10x Genomics) with previously published single cell RNA-sequencing data (Hermann et al., 2018).

### Generation of *Fam71f1*- and *Fam71f2*-mutant mice

Both *Fam71f1*- and *Fam71f2*-mutant mice were generated by using the CRISPR/Cas9 system. The gRNA design and off-target analysis were performed using CRISPRdirect software (http://crispr.dbcls.jp/) (Naito et al., 2015). gRNA sequences of each gene are listed in Table S3. Fertilized eggs were obtained from the oviducts of superovulated B6D2F1 females mated with B6D2F1 males. For *Fam71f1*-deficient mice, a pX330 plasmid (42230, Addgene) carrying the target gRNA sequence was microinjected into the pronuclei of zygotes at 5 ng/μl. For *Fam71f2*-deficient mice, ribonucleoprotein complexes containing synthesized CRISPR RNA (crRNA) (Sigma-Aldrich), *trans*-activating crRNA (tracrRNA) (TRACRRNA05N-5NMOL, Sigma-Aldrich) and CAS9 protein (A36497, Thermo Fisher Scientific) were introduced into fertilized eggs using a NEPA21 super electroporator (NEPA GENE) (Noda et al., 2019). The treated eggs were cultivated in potassium simplex optimization medium (KSOM) to the two-cell stage and then transplanted into the oviducts of 0.5 day pseudopregnant ICR females. The pups were obtained by natural delivery or Cesarean section and mutant alleles were confirmed by PCR and subsequent Sanger sequencing. Genotyping PCR of subsequent generations was performed using KOD FX Neo. The primers for each gene are listed in Table S2. For the *Fam71f1*-mutant line, the PCR product was digested by RsaI restriction enzyme to confirm the mutant allele.

### Generation of *Fam71f1* transgenic mutant mice

The *Fam71f1* transgene included C-terminal PA-tagged mouse *Fam71f1* cDNA with a rabbit polyA signal under the control of the mouse *Clgn* promoter (Fig. S5A). The linearized DNA was microinjected into the pronucleus of fertilized eggs, cultivated to the two-cell stage and then transplanted into the oviducts of pseudopregnant ICR females as mentioned above. The presence of the Tg allele was verified by PCR. The primers used are listed in Table S2.

### *In vivo* male fertility check

Each control (*Fam71f1^+/−4^* or wild-type) or mutant male was caged with two or three 8-week-old B6D2F1 female mice for 2 months. Plugs and the number of pups were checked every morning. At least three males were used for each group.

### Morphological analysis of testis and spermatozoa

Male mice (12-18 weeks old) were euthanized and testes and cauda epididymis were dissected. Gross morphology of each testis was observed using a SZX7 stereomicroscope system (Olympus). Cauda epididymal spermatozoa were dispersed in PBS (14190144, Thermo Fisher Scientific) and observed using a Nikon Eclipse Ti microscope connected to a Nikon C2 confocal module.

### *In vitro* fertilization

*In vitro* fertilization was performed as described previously (Tokuhiro et al., 2012). Cauda epididymal spermatozoa were dispersed in a drop of Toyoda, Yokoyama, Hoshi (TYH) medium (Toyoda et al., 1971) covered with paraffin oil for 2 h at 37°C under 5% CO_2_ for capacitation. Eggs obtained from the oviducts of superovulated females were placed in a TYH drop. To remove the cumulus cells, eggs were treated with 330 µg/ml of hyaluronidase (FUJIFILM Wako Pure Chemical Cor.) for 5 min. To remove the ZP, eggs were treated with 1 mg/ml of collagenase (C1639, Sigma-Aldrich) for 5 min. The capacitated spermatozoa were then added to the drop containing cumulus-intact, cumulus-free, ZP-free eggs at a final concentration of 2×10^5^ spermatozoa/ml. After 8 h of insemination, the formation of pronuclei was observed.

### Sperm velocity analysis

Sperm velocity was analyzed as described previously (Miyata et al., 2015; Morohoshi et al., 2020). Cauda epididymal spermatozoa were dispersed in a drop of TYH medium. Sperm velocity was measured using the CEROS sperm analysis system (software version 12.3; Hamilton Thorne Biosciences) for *Fam71f1* or CEROS II sperm analysis system (software version 1.4; Hamilton Thorne Biosciences) for *Fam71f2* at 10 min and 2 h after incubation at 37°C under 5% CO_2_. More than 200 spermatozoa were analyzed for each male.

### Acrosome reaction analysis with IZUMO1 staining

Cauda epididymal spermatozoa were dispersed in a drop of TYH medium. After 5 min or 4 h incubation at 37°C under 5% CO_2_, anti-IZUMO1 antibodies, Alexa Fluor 488-conjugated secondary antibodies, and 10 µg/ml of propidium iodide (PI) were added and incubated for 10 min. To induce the acrosome reaction with Ca^2+^ ionophore A23187, 20 μM A23187 (100106, MERCK) was added after 4 h incubation and incubated for 10 min before IZUMO1 staining. An aliquot of the spermatozoa suspension was then placed on a glass slide and observed under a BX-53 microscope (Olympus). The antibodies and dilutions used are listed in Table S4.

### Acrosome reaction analysis using *Acr*-*EGFP* transgenic mice

The rate of acrosome reaction was analyzed as described previously (Nakanishi et al., 1999) The *Fam71f1*-mutant line was crossed with B6D2 Tg mice carrying *CAG/Su9-DsRed2*, *Acr3-EGFP* (Red Body Green Sperm; RBGS). Cauda epididymal spermatozoa were dispersed in a drop of TYH medium. After 10 min or 4 h incubation at 37°C under 5% CO_2_, 10 µg/ml of PI was added to the drop and, subsequently, an aliquot of the sperm suspension was placed on a glass slide; the acrosome reaction was determined by observing EGFP signals while distinguishing viable cells with PI staining with a BX-53 microscope (Olympus). To induce the acrosome reaction with Ca^2+^ ionophore A23187, 20 μM A23187 (100106, MERCK) was added after 4 h incubation. After 10 min of incubation, PI was also added and the acrosome reaction was analyzed as described above.

### Construction of expression plasmids

cDNAs encoding *Fam71f1*, *Rab2a* and *Rab2b* were amplified from mouse testis (C57BL/6N). *Fam71f1* cDNA was cloned into FLAG-tagged, PA-tagged, or HA-tagged (C terminus) pCAG vectors that contain the CAG promoter and a rabbit globin poly (A) signal (Niwa et al., 1991). *Rab2a* and *Rab2b* cDNAs were cloned into the PA-tagged (N terminus) pCAG vector. CA and CN mutants of *Rab2a* were generated using this amplicon by a KOD plus mutagenesis kit (TOYOBO) following the manufacturer's protocol. pEF-*FLAG-Rab2B* (CA/CN) vectors (Aizawa and Fukuda, 2015) were kindly provided by Dr Mitsunori Fukuda (Graduate School of Life Sciences, Tohoku University, Tohoku, Japan). Primers used to construct these plasmids are listed in Table S2.

### Cell culture and transfection

HEK293T (Tiscornia et al., 2006) and COS-7 cells (RCB0539, RIKEN BioResource Research Center) were cultured in DMEM (11995–065, Thermo Fisher Scientific) supplemented with 10% fetal bovine serum (S1560, Biowest) and 1% penicillin-streptomycin-glutamine (10378–016, Thermo Fisher Scientific) at 37°C under 5% CO_2_. For HEK293T cells, plasmid DNA were transiently transfected (Tiscornia et al., 2006) and cultured for 48 h. For COS-7 cells, PEI MAX reagent (Polyscience) was used for transient transfection, followed by culturing for 24 h.

### Protein extraction from testis, spermatozoa and cultured cells

Testis, spermatozoa from cauda epididymis, and harvested cells were suspended in lysis buffer [1% Triton X-100, 50 mM Tris-HCl pH 7.5, 150 mM NaCl] with protease inhibitor cocktail (25955, Nacalai Tesque) and incubated for 1 h on ice. The lysate was centrifuged at 15,300 ***g*** for 15 min at 4°C. The obtained supernatants were subjected to immunoprecipitation or SDS-PAGE for immunoblotting. PNGase F (P0704S, New England Biolabs Japan) or *O*-glycosidase (P0733S, New England Biolabs Japan) were used to treat testis and sperm lysates with glycosidases, following the manufacturer's protocol.

### Immunoprecipitation and immunoblotting

The supernatants derived from testis, spermatozoa and cultured cells were incubated for 1 h at 4°C with antibody-conjugated Dynabeads Protein G (10009D, Thermo Fisher Scientific). After two or three washes with mild buffer (40 mM Tris-HCl pH 7.5, 150 mM NaCl, 0.1% Triton X-100 and 10% glycerol), the immune complexes were eluted with sample buffer with 2-mercaptoethanol (for immunoblotting) or elution buffer (1858606, Thermo Fisher Scientific, for mass spectrometry). Immunoblotting was performed as previously described (Morohoshi et al., 2020). Antibodies used are listed in Table S4.

### Immunocytochemistry and immunohistochemistry

For staining fixed spermatozoa, spermatozoa collected from the cauda epididymis were diluted in PBS, spread onto glass slides, and incubated at 37°C until dry. The samples were fixed with 4% paraformaldehyde in PBS (hereafter 4% PFA) for 20 min at room temperature. After three 10 min washes with 0.05% Tween 20 in PBS (hereafter PBS-T), the slides were blocked with 5% BSA and 10% goat serum in PBS for 1 h at room temperature and incubated with primary antibodies overnight at 4°C. After three 10 min washes with PBS-T, the slides were incubated with secondary antibodies or Alexa Fluor 488 conjugated-lectin PNA From *Arachis hypogaea* (peanut) (L21409, Thermo Fisher Scientific) at room temperature for 90 min, and then washed with PBS-T three times for 10 min each. To stain the nucleus, the slides were incubated with Hoechst 33342 (1:10,000; H3570, Thermo Fisher Scientific) for 5 min, washed with PBS-T three times for 10 min each, and then mounted using Immu-Mount (9990402, Thermo Fisher Scientific).

For staining testis sections, testes were dissected and fixed overnight at 4°C with 4% PFA. The samples were dehydrated, embedded in paraffin and sectioned at a thickness of 5 µm. Paraffin sections were rehydrated, boiled in antigen retrieval buffer (1× citrate buffer, pH 6.0; ab93678, Abcam) for 15 min using a microwave. After two washes with ultrapure water and two washes with PBS, the slides were blocked with 3% goat serum for 1 h at room temperature and incubated with primary antibodies overnight at 4°C. Subsequent steps were performed in the same way as for staining fixed spermatozoa, except that PBS was used instead of PBS-T for washing.

For staining COS-7 cells, cells on coverslips were fixed with 4% PFA for 10 min at room temperature. After three washes with PBS, the cells were permeabilized with 0.1% Triton X-100 in PBS for 20 min, blocked with 1% BSA for 1 h at room temperature, and incubated with primary antibodies overnight at 4°C. Subsequent steps are performed in the same way as for staining spermatozoa.

All samples were observed using a Nikon Eclipse Ti microscope connected to a Nikon C2 confocal module. Fluorescence images were false-colored and cropped using Fiji software (version 2.1.0, NIH). Antibodies used are listed in Table S4.

### Ultrastructural analysis using transmission electron microscopy

Testis and cauda epididymis samples were prepared as previously described (Shimada et al., 2019). The ultrathin sections were observed using a JEM-1400 plus electron microscope (JEOL) at 80 kV with a CCD Veleta 2K×2K camera (Olympus).

### Ultrastructural analysis using scanning electron microscopy

Cauda epididymal spermatozoa were dispersed in a drop of TYH medium and transferred to 2 ml round-bottom tubes containing PBS. After two washes with PBS, spermatozoa were mounted on coverslips and fixed with 1% glutaraldehyde in PBS on ice. Subsequent specimens were prepared as previously described (Shimada et al., 2021). The specimens were observed using a S-4800 field emission scanning electron microscope (Hitachi).

### Immunoprecipitation-mass spectrometry

Immunoprecipitated samples were subjected to mass spectrometry analysis as previously described (Shimada et al., 2021). The resulting proteins were identified using Mascot (version: 2.7.0, Matrix Science) by referring to Scaffold_4.10.0 (Proteome Software). Human keratin peptides were removed from the result.

### Statistical analysis

Statistical analyses were performed using a two-tailed Student's *t*-test (*n*≥3) using R (version 4.0.2) via RStudio (version 1.3.1073). Differences were considered significant at *P<0.05, **P<0.01 and ***P<0.001. Data represent the mean±standard deviation (s.d.); error bars indicate s.d.

## Supplementary Material

Supplementary information

Reviewer comments

## References

[DEV199644C1] Aizawa, M. and Fukuda, M. (2015). Small GTPase Rab2B and Its specific binding protein Golgi-associated rab2b interactor-like 4 (GARI-l4) regulate Golgi morphology. *J. Biol. Chem.* 290, 22250-22261. 10.1074/jbc.M115.669242 26209634 PMC4571976

[DEV199644C2] Battaglia, D. E., Koehler, J. K., Klein, N. A. and Tucker, M. J. (1997). Failure of oocyte activation after intracytoplasmic sperm injection using round-headed sperm. *Fertil. Steril.* 68, 118-122. 10.1016/S0015-0282(97)81486-0 9207595

[DEV199644C3] Berruti, G. and Paiardi, C. (2011). Acrosome biogenesis. *Spermatogenesis* 1, 95-98. 10.4161/spmg.1.2.16820 22319656 PMC3271650

[DEV199644C4] Buffone, M. G., Foster, J. A. and Gerton, G. L. (2008). The role of the acrosomal matrix in fertilization. *Int. J. Dev. Biol.* 52, 511-522. 10.1387/ijdb.072532mb 18649264

[DEV199644C5] Celse, T., Cazin, C., Mietton, F., Martinez, G., Martinez, D., Thierry-Mieg, N., Septier, A., Guillemain, C., Beurois, J., Clergeau, A. et al. (2021). Genetic analyses of a large cohort of infertile patients with globozoospermia, DPY19L2 still the main actor, GGN confirmed as a guest player. *Hum. Genet.* 140, 43-57. 10.1007/s00439-020-02229-0 33108537

[DEV199644C6] Coutton, C., Zouari, R., Abada, F., Khelifa, M. B., Merdassi, G., Triki, C., Escalier, D., Hesters, L., Mitchell, V., Levy, R. et al. (2012). MLPA and sequence analysis of DPY19L2 reveals point mutations causing globozoospermia. *Hum. Reprod.* 27, 2549-2558. 10.1093/humrep/des160 22627659

[DEV199644C7] Coutton, C., Escoffier, J., Martinez, G., Arnoult, C. and Ray, P. F. (2015). Teratozoospermia: Spotlight on the main genetic actors in the human. *Hum. Reprod. Update* 21, 455-485. 10.1093/humupd/dmv020 25888788

[DEV199644C8] Dam, A. H. D. M., Feenstra, I., Westphal, J. R., Ramos, L., van Golde, R. J. T. and Kremer, J. A. M. (2007a). Globozoospermia revisited. *Hum. Reprod. Update* 13, 63-75. 10.1093/humupd/dml047 17008355

[DEV199644C9] Dam, A. H. D. M., Koscinski, I., Kremer, J. A. M., Moutou, C., Jaeger, A.-S., Oudakker, A. R., Tournaye, H., Charlet, N., Lagier-Tourenne, C., van Bokhoven, H. et al. (2007b). Homozygous mutation in SPATA16 is associated with male infertility in human globozoospermia. *Am. J. Hum. Genet.* 81, 813-820. 10.1086/521314 17847006 PMC2227931

[DEV199644C10] Fujihara, Y., Satouh, Y., Inoue, N., Isotani, A., Ikawa, M. and Okabe, M. (2012). SPACA1-deficient male mice are infertile with abnormally shaped sperm heads reminiscent of globozoospermia. *Development* 139, 3583-3589. 10.1242/dev.081778 22949614

[DEV199644C11] Fujihara, Y., Lu, Y., Noda, T., Oji, A., Larasati, T., Kojima-Kita, K., Yu, Z., Matzuk, R. M., Matzuk, M. M. and Ikawa, M. (2020). Spermatozoa lacking fertilization influencing membrane protein (FIMP) fail to fuse with oocytes in mice. *Proc. Natl. Acad. Sci. USA* 117, 9393-9400. 10.1073/pnas.1917060117 32295885 PMC7196805

[DEV199644C12] Fukuda, M., Kanno, E., Ishibashi, K. and Itoh, T. (2008). Large scale screening for novel rab effectors reveals unexpected broad Rab binding specificity. *Mol. Cell. Proteomics* 7, 1031-1042. 10.1074/mcp.M700569-MCP200 18256213

[DEV199644C13] Guidi, L. G., Holloway, Z. G., Arnoult, C., Ray, P. F., Monaco, A. P., Molnár, Z. and Velayos-Baeza, A. (2018). AU040320 deficiency leads to disruption of acrosome biogenesis and infertility in homozygous mutant mice. *Sci. Rep.* 8, 10379. 10.1038/s41598-018-28666-6 29991750 PMC6039479

[DEV199644C14] Harbuz, R., Zouari, R., Pierre, V., Ben Khelifa, M., Kharouf, M., Coutton, C., Merdassi, G., Abada, F., Escoffier, J., Nikas, Y. et al. (2011). A recurrent deletion of DPY19L2 causes infertility in man by blocking sperm head elongation and acrosome formation. *Am. J. Hum. Genet.* 88, 351-361. 10.1016/j.ajhg.2011.02.007 21397064 PMC3059422

[DEV199644C15] Hermann, B. P., Cheng, K., Singh, A., Roa-De La Cruz, L., Mutoji, K. N., Chen, I.-C., Gildersleeve, H., Lehle, J. D., Mayo, M., Westernströer, B. et al. (2018). The mammalian spermatogenesis single-cell transcriptome, from spermatogonial stem cells to spermatids. *Cell Rep.* 25, 1650-1667.e8. 10.1016/j.celrep.2018.10.026 30404016 PMC6384825

[DEV199644C16] Ikawa, M., Nakanishi, T., Yamada, S., Wada, I., Kominami, K., Tanaka, H., Nozaki, M., Nishimune, Y. and Okabe, M. (2001). Calmegin is required for fertilin α/β heterodimerization and sperm fertility. *Dev. Biol.* 240, 254-261. 10.1006/dbio.2001.0462 11784061

[DEV199644C17] Inoue, N., Ikawa, M., Isotani, A. and Okabe, M. (2005). The immunoglobulin superfamily protein Izumo is required for sperm to fuse with eggs. *Nature* 434, 234-238. 10.1038/nature03362 15759005

[DEV199644C18] Jan, S. Z., Hamer, G., Repping, S., de Rooij, D. G., van Pelt, A. M. M. and Vormer, T. L. (2012). Molecular control of rodent spermatogenesis. *Biochim. Biophys. Acta Mol. Basis Dis.* 1822, 1838-1850. 10.1016/j.bbadis.2012.02.008 22366765

[DEV199644C19] Kajiho, H., Kajiho, Y., Frittoli, E., Confalonieri, S., Bertalot, G., Viale, G., Di Fiore, P. P., Oldani, A., Garre, M., Beznoussenko, G. V. et al. (2016). RAB2A controls MT1-MMP endocytic and E-cadherin polarized Golgi trafficking to promote invasive breast cancer programs. *EMBO Rep.* 17, 1061-1080. 10.15252/embr.201642032 27255086 PMC4931572

[DEV199644C20] Käll, L., Krogh, A. and Sonnhammer, E. L. L. (2007). Advantages of combined transmembrane topology and signal peptide prediction--the Phobius web server. *Nucleic Acids Res.* 35, W429-W432. 10.1093/nar/gkm256 17483518 PMC1933244

[DEV199644C21] Kang-Decker, N., Mantchev, G. T., Juneja, S. C., McNiven, M. A. and van Deursen, J. M. (2001). Lack of acrosome formation in Hrb-deficient mice. *Science* 294, 1531-1533. 10.1126/science.1063665 11711676

[DEV199644C22] Karaca, N., Yilmaz, R., Kanten, G. E., Kervancioglu, E., Solakoglu, S. and Kervancioglu, M. E. (2014). First successful pregnancy in a globozoospermic patient having homozygous mutation in SPATA16. *Fertil. Steril.* 102, 103-107. 10.1016/j.fertnstert.2014.04.002 24825417

[DEV199644C23] Khawar, M. B., Gao, H. and Li, W. (2019). Mechanism of Acrosome Biogenesis in Mammals. *Front. Cell Dev. Biol.* 7, 1-12. 10.3389/fcell.2019.00195 31620437 PMC6759486

[DEV199644C24] Kluin, P. M., Kramer, M. F. and de Rooij, D. G. (1982). Spermatogenesis in the immature mouse proceeds faster than in the adult. *Int. J. Androl.* 5, 282-294. 10.1111/j.1365-2605.1982.tb00257.x 7118267

[DEV199644C25] Koscinski, I., Elinati, E., Fossard, C., Redin, C., Muller, J., Velez De La Calle, J., Schmitt, F., Ben Khelifa, M., Ray, P., Kilani, Z. et al. (2011). DPY19L2 deletion as a major cause of globozoospermia. *Am. J. Hum. Genet.* 88, 344-350. 10.1016/j.ajhg.2011.01.018 21397063 PMC3059416

[DEV199644C26] Lin, Y.-N., Roy, A., Yan, W., Burns, K. H. and Matzuk, M. M. (2007). Loss of zona pellucida binding proteins in the acrosomal matrix disrupts acrosome biogenesis and sperm morphogenesis. *Mol. Cell. Biol.* 27, 6794-6805. 10.1128/MCB.01029-07 17664285 PMC2099232

[DEV199644C27] Liu, G., Shi, Q.-W. and Lu, G.-X. (2010). A newly discovered mutation in PICK1 in a human with globozoospermia. *Asian J. Androl.* 12, 556-560. 10.1038/aja.2010.47 20562896 PMC3739375

[DEV199644C28] Lörincz, P., Tóth, S., Benkö, P., Lakatos, Z., Boda, A., Glatz, G., Zobel, M., Bisi, S., Hegedüs, K., Takáts, S. et al. (2017). Rab2 promotes autophagic and endocytic lysosomal degradation. *J. Cell Biol.* 216, 1937-1947. 10.1083/jcb.201611027 28483915 PMC5496615

[DEV199644C29] Malcher, A., Rozwadowska, N., Stokowy, T., Kolanowski, T., Jedrzejczak, P., Zietkowiak, W. and Kurpisz, M. (2013). Potential biomarkers of nonobstructive azoospermia identified in microarray gene expression analysis. *Fertil. Steril.* 100, 1686-1694.e7. 10.1016/j.fertnstert.2013.07.1999 24012201

[DEV199644C30] Mashiko, D., Fujihara, Y., Satouh, Y., Miyata, H., Isotani, A. and Ikawa, M. (2013). Generation of mutant mice by pronuclear injection of circular plasmid expressing Cas9 and single guided RNA. *Sci. Rep.* 3, 3355. 10.1038/srep03355 24284873 PMC3842082

[DEV199644C31] Méndez, J. and Stillman, B. (2000). Chromatin association of human origin recognition complex, cdc6, and minichromosome maintenance proteins during the cell cycle: assembly of prereplication complexes in late mitosis. *Mol. Cell. Biol.* 20, 8602-8612. 10.1128/MCB.20.22.8602-8612.2000 11046155 PMC102165

[DEV199644C32] Miyata, H., Satouh, Y., Mashiko, D., Muto, M., Nozawa, K., Shiba, K., Fujihara, Y., Isotani, A., Inaba, K. and Ikawa, M. (2015). Sperm calcineurin inhibition prevents mouse fertility with implications for male contraceptive. *Science.* 350, 442-445. 10.1126/science.aad0836 26429887

[DEV199644C33] Moreno, R. D. and Alvarado, C. P. (2006). The mammalian acrosome as a secretory lysosome: New and old evidence. *Mol. Reprod. Dev.* 73, 1430-1434. 10.1002/mrd.20581 16894549

[DEV199644C34] Morohoshi, A., Miyata, H., Shimada, K., Nozawa, K., Matsumura, T., Yanase, R., Shiba, K., Inaba, K. and Ikawa, M. (2020). Nexin-Dynein regulatory complex component DRC7 but not FBXL13 is required for sperm flagellum formation and male fertility in mice. *PLoS Genet.* 16, 1-21. 10.1371/journal.pgen.1008585 PMC6994161 31961863

[DEV199644C35] Naito, Y., Hino, K., Bono, H. and Ui-Tei, K. (2015). CRISPRdirect: Software for designing CRISPR/Cas guide RNA with reduced off-target sites. *Bioinformatics* 31, 1120-1123. 10.1093/bioinformatics/btu743 25414360 PMC4382898

[DEV199644C36] Nakanishi, T., Ikawa, M., Yamada, S., Parvinen, M., Baba, T., Nishimune, Y. and Okabe, M. (1999). Real-time observation of acrosomal dispersal from mouse sperm using GFP as a marker protein. *FEBS Lett.* 449, 277-283. 10.1016/S0014-5793(99)00433-0 10338148

[DEV199644C37] Niwa, H., Yamamura, K. and Miyazaki, J. (1991). Efficient selection for high-expression transfectants with a novel eukaryotic vector. *Gene* 108, 193-199. 10.1016/0378-1119(91)90434-D 1660837

[DEV199644C38] Noda, T., Sakurai, N., Nozawa, K., Kobayashi, S., Devlin, D. J., Matzuk, M. M. and Ikawa, M. (2019). Nine genes abundantly expressed in the epididymis are not essential for male fecundity in mice. *Andrology* 7, 644-653. 10.1111/andr.12621 30927342 PMC6688925

[DEV199644C39] Noda, T., Lu, Y., Fujihara, Y., Oura, S., Koyano, T., Kobayashi, S., Matzuk, M. M. and Ikawa, M. (2020). Sperm proteins SOF1, TMEM95, and SPACA6 are required for sperm-oocyte fusion in mice. *Proc. Natl. Acad. Sci. USA* 117, 11493-11502. 10.1073/pnas.1922650117 32393636 PMC7261011

[DEV199644C40] Okabe, M. (2013). The cell biology of mammalian fertilization. *Development* 140, 4471-4479. 10.1242/dev.090613 24194470

[DEV199644C41] Paiardi, C., Pasini, M. E., Gioria, M. and Berruti, G. (2011). Failure of acrosome formation and globozoospermia in the wobbler mouse, a Vps54 spontaneous recessive mutant. *Spermatogenesis* 1, 52-62. 10.4161/spmg.1.1.14698 21866276 PMC3158644

[DEV199644C42] Pierre, V., Martinez, G., Coutton, C., Delaroche, J., Yassine, S., Novella, C., Pernet-Gallay, K., Hennebicq, S., Ray, P. F. and Arnoult, C. (2012). Absence of Dpy19l2, a new inner nuclear membrane protein, causes globozoospermia in mice by preventing the anchoring of the acrosome to the nucleus. *Development* 139, 2955-2965. 10.1242/dev.077982 22764053

[DEV199644C43] Ramalho-Santos, J. and Moreno, R. D. (2001). Targeting and fusion proteins during mammalian spermiogenesis. *Biol. Res.* 34, 147-152. 10.4067/S0716-97602001000200021 11715208

[DEV199644C44] Ramalho-Santos, J., Moreno, R. D., Wessel, G. M., Chan, E. K. L. and Schatten, G. (2001). Membrane trafficking machinery components associated with the mammalian acrosome during spermiogenesis. *Exp. Cell Res.* 267, 45-60. 10.1006/excr.2000.5119 11412037

[DEV199644C45] Satouh, Y., Inoue, N., Ikawa, M. and Okabe, M. (2012). Visualization of the moment of mouse sperm-egg fusion and dynamic localization of IZUMO1. *J. Cell Sci.* 125, 4985-4990. 10.1242/jcs.100867 22946049

[DEV199644C46] Shimada, K., Kato, H., Miyata, H. and Ikawa, M. (2019). Glycerol kinase 2 is essential for proper arrangement of crescent-like mitochondria to form the mitochondrial sheath during mouse spermatogenesis. *J. Reprod. Dev.* 65, 155-162. 10.1262/jrd.2018-136 30662012 PMC6473107

[DEV199644C47] Shimada, K., Park, S., Miyata, H., Yu, Z., Morohoshi, A., Oura, S., Matzuk, M. M. and Ikawa, M. (2021). ARMC12 regulates spatiotemporal mitochondrial dynamics during spermiogenesis and is required for male fertility. *Proc. Natl. Acad. Sci. USA* 118, e2018355118. 10.1073/pnas.2018355118 33536340 PMC8017931

[DEV199644C48] Sievers, F., Wilm, A., Dineen, D., Gibson, T. J., Karplus, K., Li, W., Lopez, R., McWilliam, H., Remmert, M., Söding, J. et al. (2011). Fast, scalable generation of high-quality protein multiple sequence alignments using Clustal Omega. *Mol. Syst. Biol.* 7, 539. 10.1038/msb.2011.75 21988835 PMC3261699

[DEV199644C49] Stanley, P. (2011). Golgi glycosylation. *Cold Spring Harb. Perspect. Biol.* 3, 1-13. 10.1101/cshperspect.a005199 PMC3062213 21441588

[DEV199644C50] Stenmark, H. (2009). Rab GTPases as coordinators of vesicle traffic. *Nat. Rev. Mol. Cell Biol.* 10, 513-525. 10.1038/nrm2728 19603039

[DEV199644C51] Takahama, M., Fukuda, M., Ohbayashi, N., Kozaki, T. and Misawa, T. (2017). The RAB2B-GARIL5 complex promotes cytosolic dna-induced innate immune responses article the RAB2B-GARIL5 complex promotes cytosolic dna-induced innate immune responses. *Cell Rep.* 20, 2944-2954. 10.1016/j.celrep.2017.08.085 28930687 PMC5614515

[DEV199644C52] Tiscornia, G., Singer, O. and Verma, I. M. (2006). Production and purification of lentiviral vectors. *Nat. Protoc.* 1, 241-245. 10.1038/nprot.2006.37 17406239

[DEV199644C53] Tokuhiro, K., Ikawa, M., Benham, A. M. and Okabe, M. (2012). Protein disulfide isomerase homolog PDILT is required for quality control of sperm membrane protein ADAM3 and male fertility [corrected]. *Proc. Natl. Acad. Sci. USA* 109, 3850-3855. 10.1073/pnas.1117963109 22357757 PMC3309714

[DEV199644C54] Toyoda, Y., Yokoyama, M. and Hosi, T. (1971). Studies on the fertilization of mouse eggs in vitro. *Jpn. J. Anim. Reprod.* 16, 152-157. 10.1262/jrd1955.16.152

[DEV199644C55] Wang, H., Wan, H., Li, X., Liu, W., Chen, Q., Wang, Y., Yang, L., Tang, H., Zhang, X., Duan, E. et al. (2014). Atg7 is required for acrosome biogenesis during spermatogenesis in mice. *Cell Res.* 24, 852-869. 10.1038/cr.2014.70 24853953 PMC4085765

[DEV199644C56] Wassarman, P. M. (1999). Mammalian fertilization: Molecular aspects of gamete adhesion, exocytosis, and fusion. *Cell* 96, 175-183. 10.1016/S0092-8674(00)80558-9 9988213

[DEV199644C57] Watanabe, D., Okabe, M., Hamajima, N., Morita, T., Nishina, Y. and Nishimune, Y. (1995). Characterization of the testis-specific gene ‘calmegin’ promoter sequence and its activity defined by transgenic mouse experiments. *FEBS Lett.* 368, 509-512. 10.1016/0014-5793(95)00729-S 7635209

[DEV199644C58] Xiao, N., Kam, C., Shen, C., Jin, W., Wang, J., Kwong, M. L., Jiang, L. and Xia, J. (2009). PICK1 deficiency causes male infertility in mice by disrupting acrosome formation. *J. Clin. Invest.* 119, 802-812. 10.1172/JCI36230 19258705 PMC2662547

[DEV199644C59] Yao, R., Ito, C., Natsume, Y., Sugitani, Y., Yamanaka, H., Kuretake, S., Yanagida, K., Sato, A., Toshimori, K. and Noda, T. (2002). Lack of acrosome formation in mice lacking a Golgi protein, GOPC. *Proc. Natl. Acad. Sci. USA* 99, 11211-11216. 10.1073/pnas.162027899 12149515 PMC123235

[DEV199644C60] Yatsenko, A. N., O'Neil, D. S., Roy, A., Arias-Mendoza, P. A., Chen, R., Murthy, L. J., Lamb, D. J. and Matzuk, M. M. (2012). Association of mutations in the zona pellucida binding protein 1 (ZPBP1) gene with abnormal sperm head morphology in infertile men. *Mol. Hum. Reprod.* 18, 14-21. 10.1093/molehr/gar057 21911476 PMC3244884

[DEV199644C61] Yildiz, Y., Matern, H., Thompson, B., Allegood, J. C., Warren, R. L., Ramirez, D. M. O., Hammer, R. E., Hamra, F. K., Matern, S. and Russell, D. W. (2006). Mutation of β-glucosidase 2 causes glycolipid storage disease and impaired male fertility. *J. Clin. Invest.* 116, 2985-2994. 10.1172/JCI29224 17080196 PMC1626112

