# Supplementary material for: FAM71F1 binds to RAB2A and RAB2B and is essential for acrosome formation and male fertility in mice
Source: Development. 2021 Oct 29;148(21):dev199644. doi: 10.1242/dev.199644 (PMC8602946; doi:10.1242/dev.199644)
Supplement: Supplementary information [file develop-148-199644-s1.pdf]

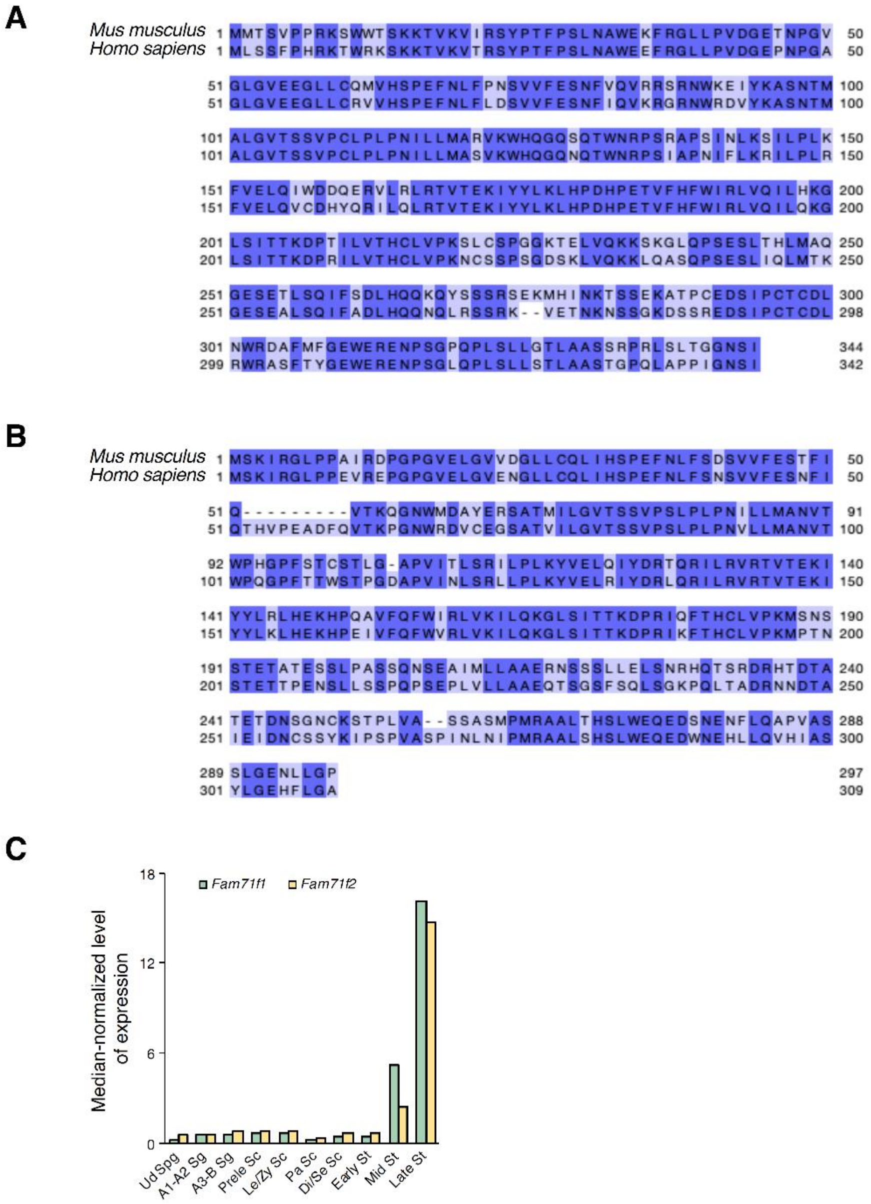

Fig. S1. Similarity of amino acid sequences and expression patterns of *Fam71f1* and *Fam71f2*.

(A) Amino acid sequence of FAM71F1 in *Mus musculus* and *Homo sapiens*. Dark purple indicates matching sequences in both species. (B) Amino acid sequence of FAM71F2 in *Mus musculus* and *Homo sapiens*. Dark purple indicates matching sequences in both species. (C) *Fam71f1* and *Fam71f2* expression during spermatogenesis in mice. Ud Spg, undifferentiated spermatogonia; A1-A2 Sg, A1-A2 differentiating spermatogonia; A3-B Sg, A3- A4-In-B differentiating spermatogonia; Prele Sc, preleptotene spermatocytes; Le/Zy Sc, leptotene/zygotene spermatocytes; Pa Sc, pachytene spermatocytes; Di/Se Sc, diplotene/secondary spermatocytes; Early St, early round spermatids; Mid St, mid round spermatids; Late St, late round spermatids.

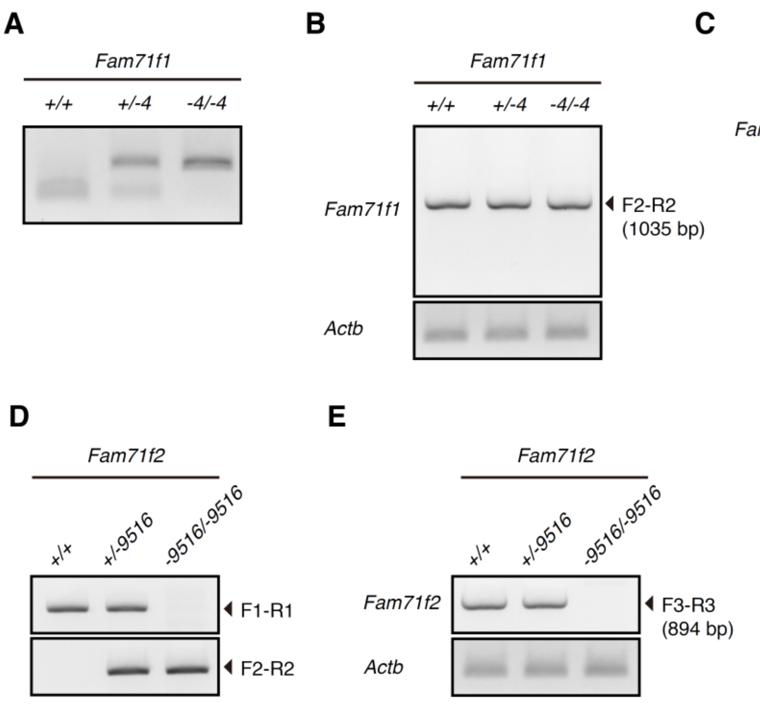

Fig. S2. Generation of *Fam71f1* or *Fam71f2* knockout mice.

(A) Genotyping of *Fam71f1* mutant mice. F1 and R1 primers in Fig. 1C were used and the PCR product was digested with RsaI. The PCR product of the wild-type allele was cut into 155 bp and 183 bp products after RsaI treatment. (B) RT-PCR analysis for *Fam71f1* expression in testes. F2 and R2 primers in Fig. 1C were used. *Actb* was used as a loading control. (C) Amino acid sequences encoded by *Fam71f1*$^{+/+}$ and *Fam71f1*$^{-4/-4}$ alleles. The 4 bp deletion in the second exon of *Fam71f1* causes a S107Y mutation and a premature stop codon. (D) Genotyping of *Fam71f2* mutant mice. F1-R1 and F2-R2 primers in Fig. 1E were used. F2 and R2 can amplify the KO allele because of the large deletion. (E) RT-PCR analysis for *Fam71f2* expression in testes. F3 and R3 primers in Fig. 1E were used. *Actb* was used as a loading control.

Development • Supplementary information

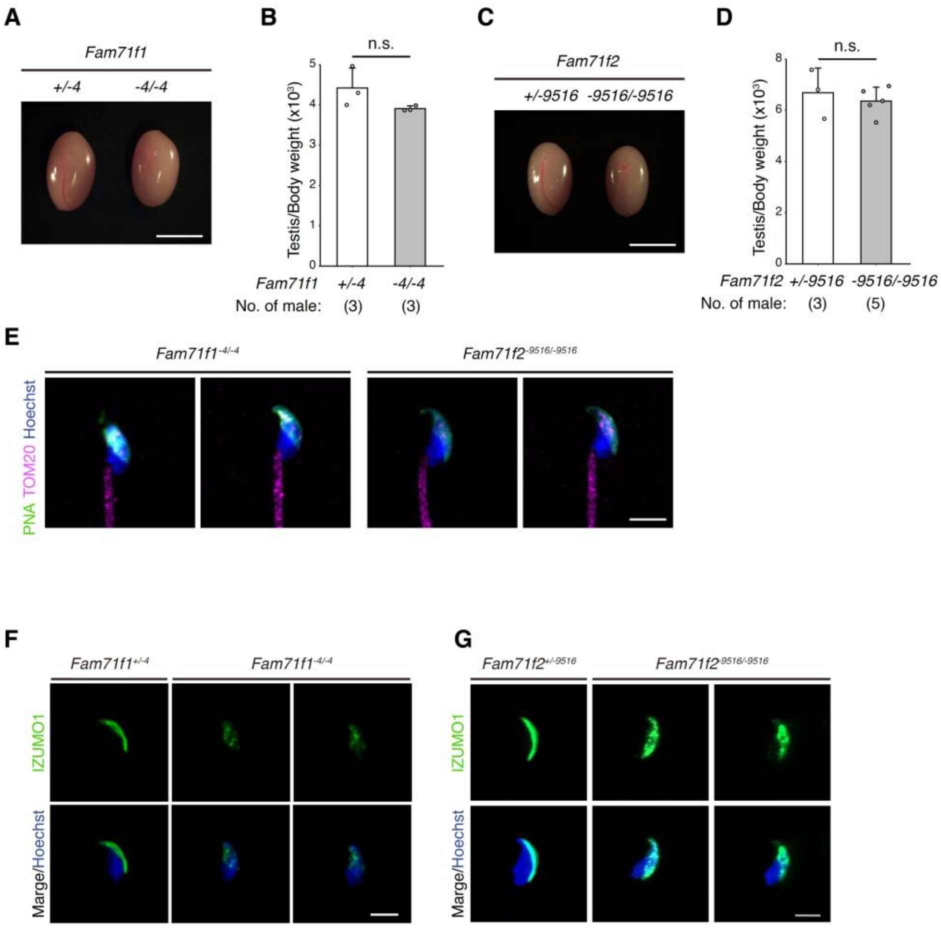

Fig. S3. Testis gross morphology and sperm head morphology in *Fam71f1* and *Fam71f2* mutant mice.

(A) Testis morphology of *Fam71f1*$^{+/-4}$ and *Fam71f1*$^{-4/-4}$ mice. Scale bar, 5 mm. (B) Testis/body weight of *Fam71f1*$^{+/-4}$ and *Fam71f1*$^{-4/-4}$ mice. Error bars represent S.D. n.s.; not-significant (unpaired Student's t-test). Number of males = 3 each. (C) Testis morphology of *Fam71f2*$^{+/-9516}$ and *Fam71f2*$^{-9516/-9516}$ mice. Scale bar, 5 mm. (D) Testis/body weight of *Fam71f2*$^{+/-9516}$ and *Fam71f2*$^{-9516/-9516}$ mice. Error bars represent S.D. n.s.; not-significant (unpaired Student's t-test). Number of males = 3 for *Fam71f2*$^{+/-9516}$ and 5 for *Fam71f2*$^{-9516/-9516}$. (E) Immunofluorescence staining of the acrosome and mitochondria. More examples of abnormal acrosome morphology (Fig. 2D) are shown. Hoechst (blue), nucleus; lectin-PNA (green), acrosome; TOM20 (magenta), mitochondria. Scale bar, 5 µm. (F) Immunofluorescence staining for IZUMO1 using fixed *Fam71f1* mutant spermatozoa. IZUMO1 was abnormally spread over the head in *Fam71f1* mutant spermatozoa. Hoechst (blue), nucleus; IZUMO1 (green). Scale bar, 5 µm. (G) Immunofluorescence staining for IZUMO1 using fixed *Fam71f2* mutant spermatozoa. IZUMO1 signal was slightly expanded in *Fam71f2* mutant spermatozoa. Hoechst (blue), nucleus; IZUMO1 (green). Scale bar, 5 µm.

Development • Supplementary information

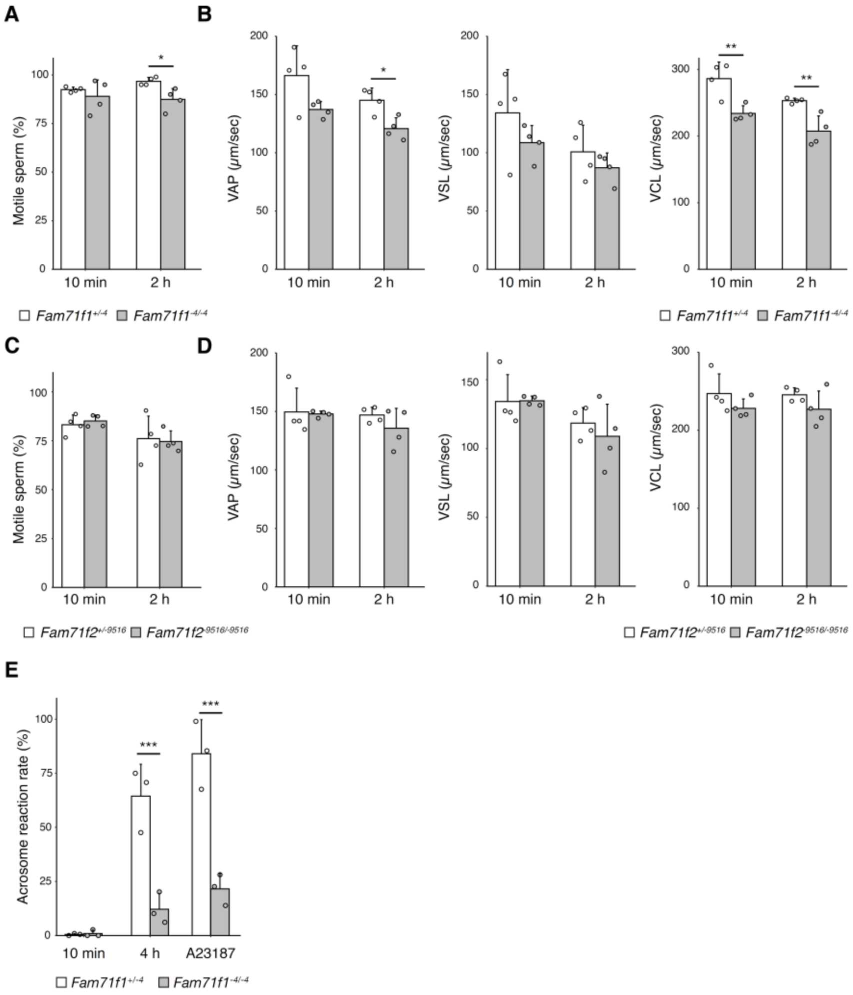

Fig. S4. Analyses of sperm motility and acrosome reaction.

(A) Percentage of motile spermatozoa in *Fam71f1* mutant males. Error bars represent S.D. *p < 0.05 (unpaired Student's t-test). Number of males = 4 each. (B) Velocity analysis of motile spermatozoa in *Fam71f1* mutant males. VAP (average path velocity), VSL (straight line velocity), and VCL (curvilinear velocity) were analyzed. *p < 0.05, **p < 0.01 (unpaired Student's t-test). Error bars represent S.D. Number of males = 4 each. (C) Percentage of motile spermatozoa in *Fam71f2* mutant males. No significant difference was found. Error bars represent S.D. Number of males = 4 each. (D) Velocity analysis of motile spermatozoa in *Fam71f2* mutant males. No significant differences were found. Error bars represent S.D. Number of males = 4 each. (E) The acrosome reaction rates at 10 minutes and 4 hours after incubation in capacitation medium. After 4 hours incubation, Ca^{2+} ionophore A23187 was added to induce the acrosome reaction. The acrosome reaction was analyzed using *Acr-EGFP* transgenic mice. After 4 hours of incubation and A23187 addition, the acrosome reaction rates were significantly lower in *Fam71f1*$^{-4/-4}$ mice compared to those of *Fam71f1*$^{+/-4}$ mice. Error bars represent S.D. ***p < 0.001 (unpaired Student's t-test). Number of males = 3 each.

Development • Supplementary information

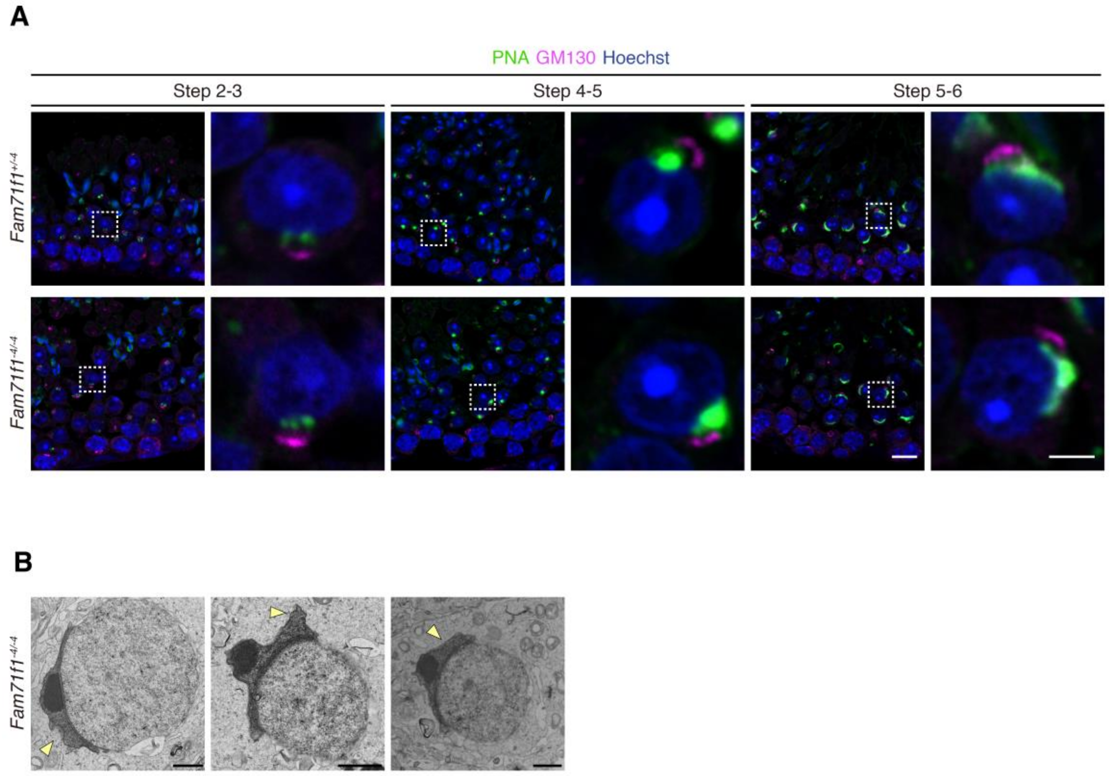

Fig. S5. Observation of the acrosome formation in *Fam71f1*$^{-4/-4}$ mice.

(A) Immunofluorescence staining of the acrosome and Golgi apparatus during spermatogenesis using testis sections. In round spermatids around step 2-6, the morphology of the acrosome was comparable to that of the control. Hoechst (blue), nucleus; lectin-PNA (green), acrosome; GM130 (magenta), Golgi apparatus. Scale bar in low magnification, 10 µm; high magnification, 5 µm. (B) TEM observation of the acrosome formation. More examples of abnormal acrosome swelling (arrowheads) around step 4-5 in *Fam71f1*$^{-4/-4}$ mice (Fig. 4B) are shown. Scale bar, 1 µm.

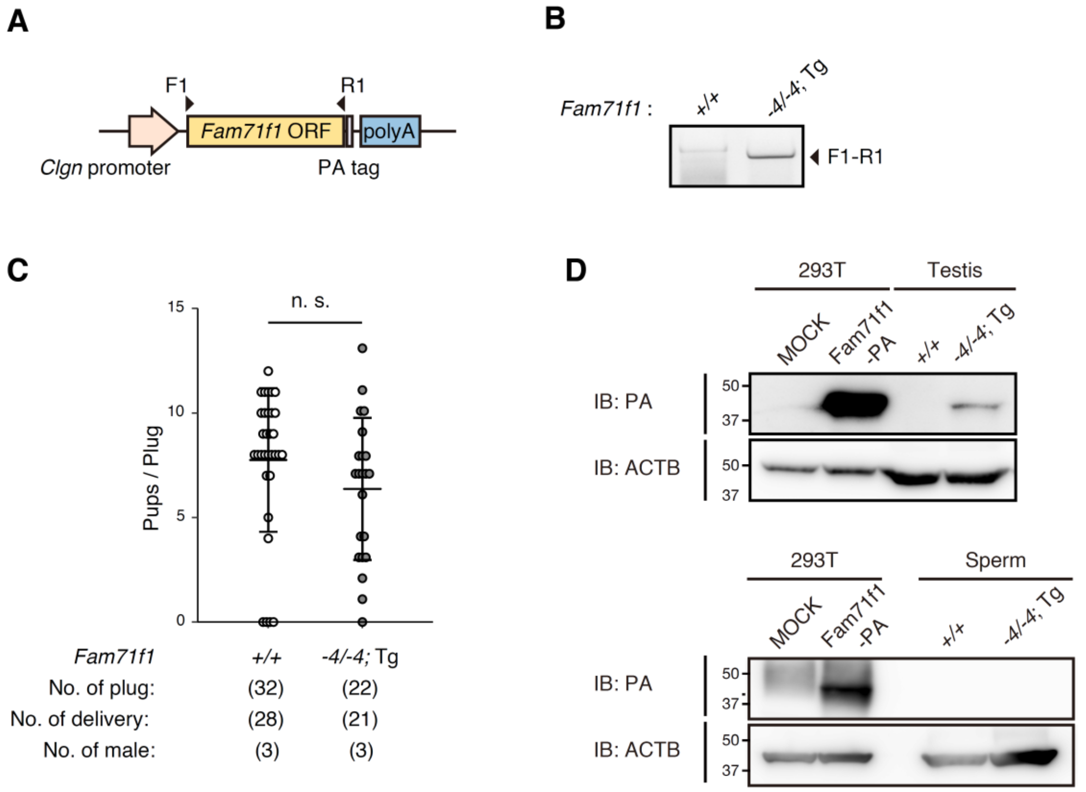

Fig. S6. Generation of *Fam71f1-PA* Tg mice.

(A) Schematic of the transgene. *Fam71f1-PA* is expressed under the *Clgn* promoter. Primers F1 and R1 are used for genotyping. (B) Genotyping of *Fam71f1-PA* Tg mice. F1 and R1 primers in Fig. S6A were used. (C) Fertility of *Fam71f1-PA* Tg mice. Males of *Fam71f1*$^{+/+}$ or *Fam71f1*$^{-4/-4}$ with the transgene were mated with wild-type females and the number of pups born per plug was counted. Error bars represent S.D. n.s.; not-significant (unpaired Student's t-test). Number of males = 3 each. (D) Immunoblotting analysis for FAM71F1-PA using HEK293T cells, testes, and cauda epididymal spermatozoa. *Fam71f1-PA* was transiently transfected in HEK293T cells for positive controls. ACTB was used as a loading control.

Development • Supplementary information

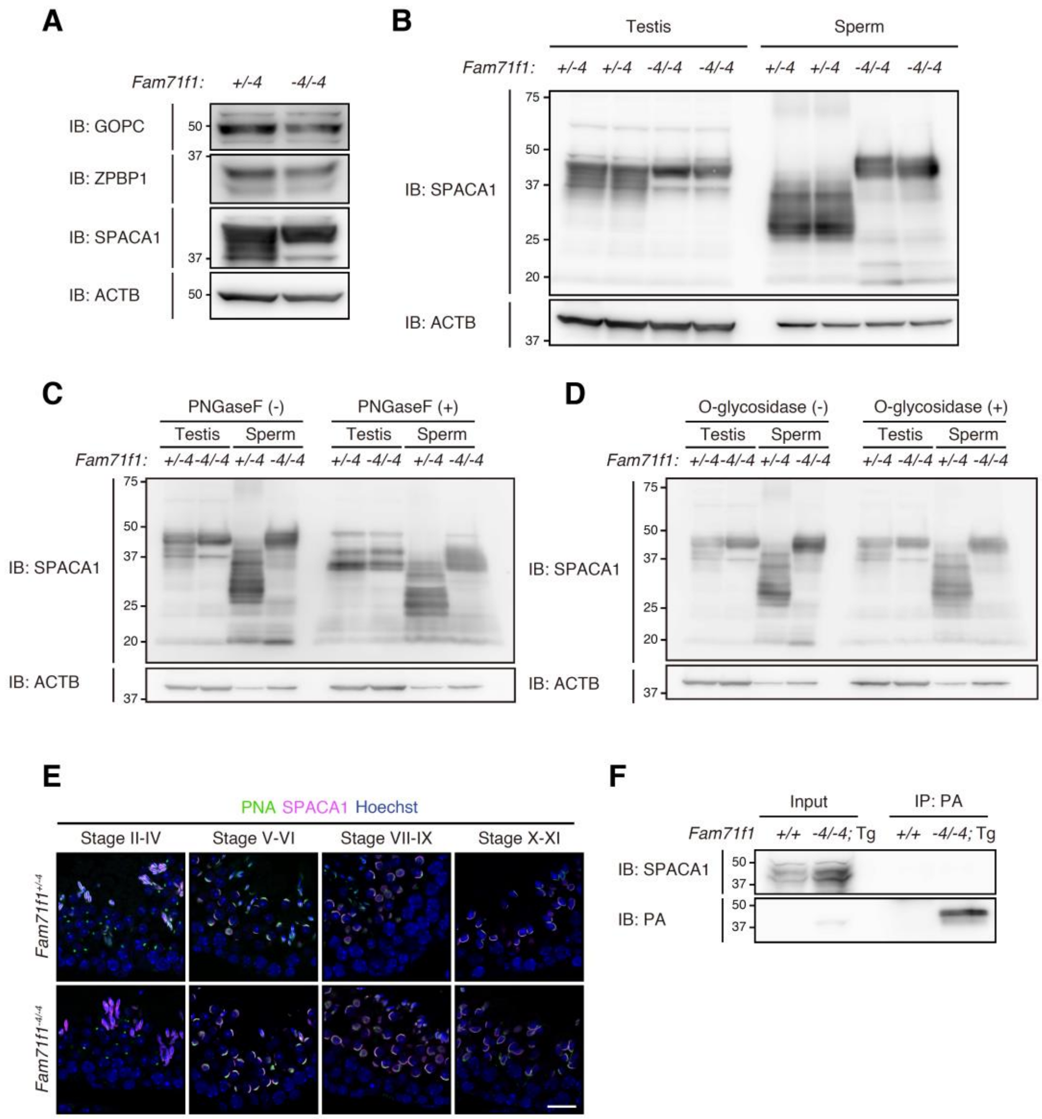

Fig. S7. Relationship between FAM71F1 and other globozoospermia-related proteins.

(A) Immunoblotting analysis of the proteins known to contribute to acrosome formation. Testis lysates from *Fam71f1*$^{+/-4}$ or *Fam71f1*$^{-4/-4}$ males were used to detect GOPC, ZPBP1, and SPACA1. ACTB was used as a loading control. (B) Immunoblotting analysis of SPACA1 in testes and cauda epididymal spermatozoa. ACTB was used as a loading control. (C) Immunoblotting analysis of SPACA1 in testes and cauda epididymal spermatozoa. Samples were treated with PNGase F. ACTB was used as a loading control. (D) Immunoblotting analysis of SPACA1 in testes and cauda epididymal spermatozoa. Samples were treated with O-glycosidase. ACTB was used as a loading control. (E) Immunofluorescence staining for the acrosome and SPACA1 during spermatogenesis using testis sections. Hoechst (blue), nucleus; lectin-PNA (green), acrosome; SPACA1 (magenta). Scale bar, 20 µm. (F) Immunoprecipitation of FAM71F1-PA using anti-PA antibody in *Fam71f1-PA* Tg testis. SPACA1 was not detected by immunoblotting analysis.

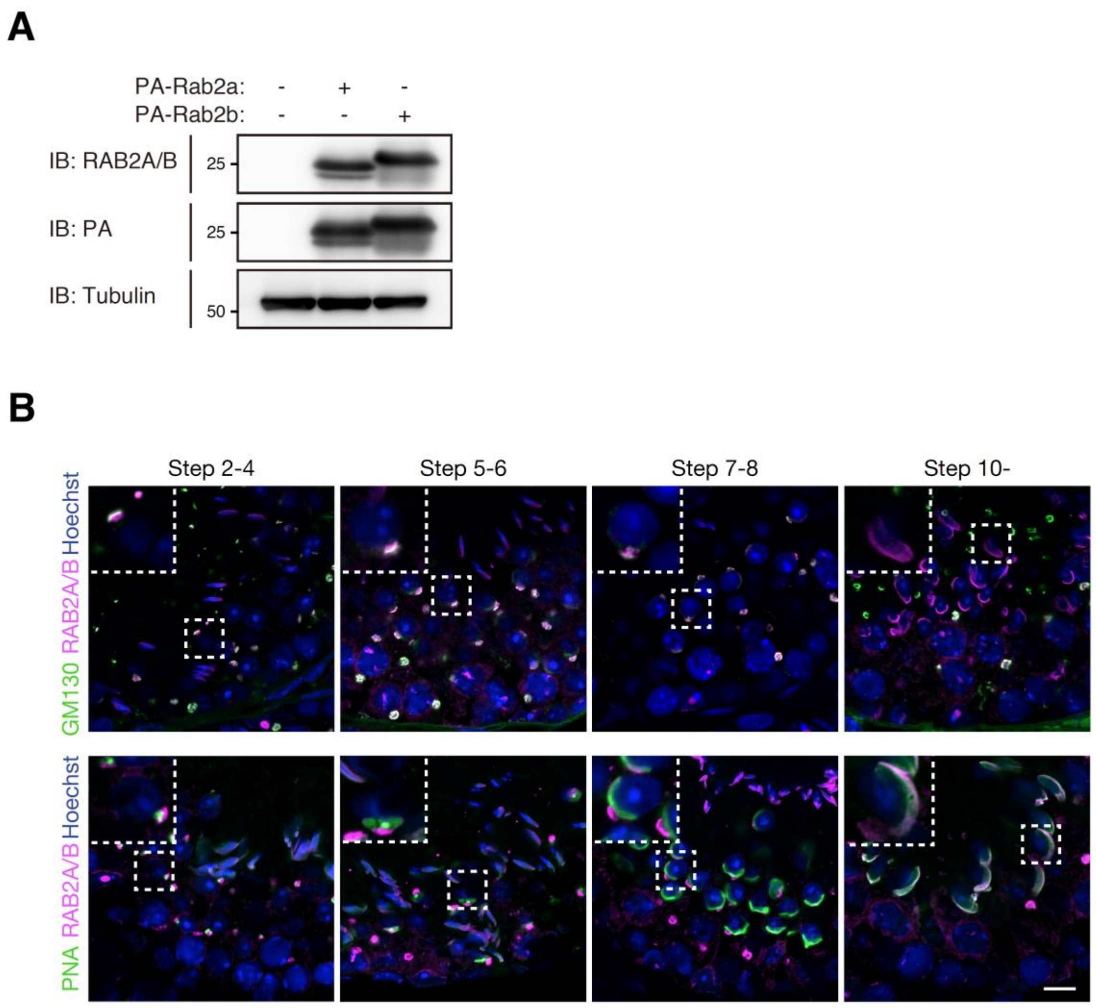

Fig. S8. Localization of RAB2A/B in the testis.

(A) Confirmation of RAB2A/B antibody. *PA-Rab2a* or *PA-Rab2b* was transiently transfected in HEK293T cells. The RAB2A/B antibody reacts with both PA-RAB2A and PA-RAB2B. Tubulin was used as a loading control. (B) Immunofluorescence staining of RAB2A/B during spermatogenesis using testis sections. RAB2A/B was localized in the Golgi apparatus until step 7-8. Enlarged pictures are shown in the top-left corner. Hoechst (blue), nucleus; GM130 (green) (upper panels), Golgi apparatus; PNA (green) (lower panels), acrosome; RAB2A/B (magenta). Scale bar, 10 µm.

Table S1. Identified proteins by immunoprecipitation - mass spectrometry.

Click here to download Table S1

Development • Supplementary information

Table S2. Sequences of primers.

Method	Target	Strand	Sequence (5'→3')	Name in Fig.
RT-PCR	*Fam71f1*	F	ATGATGACATCAGTTCCACCTAGAAAGTC	F2
		R	TATAGAGTTTCCTCCAGTTAGGGACAGCC	R2
	Fam71f2	F	ATGAGTAAAATTAGGGGCCTCCCTCC	F3
		R	GGGTCCCAACAAGTTCTCTCC	R3
	Actb	F	CATCCGTAAAGACCTCTATGCCAAC	
		R	ATGGAGCCACCGATCCACA	
Genotyping	*Fam71f1*	F	AGTGCTGAGTGAAGAAACCC	F1
		R	ATTTAACAGGTAGCCTCCCC	R1
	Fam71f1-PA tg	F	ATGATGACATCAGTTCCACCTAGAAAGTC	F1
		R	TATAGAGTTTCCTCCAGTTAGGGACAGCC	R1
	Fam71f2	F	CAGCAATGGTGATTGATGGG	F1
		R	ATCCCACTGTATTGTTCAGG	R1
		F	GTCTTTAAACTCCTATCAGG	F2
		R	CTGTGTAGCTCCTGTTTTGG	R2
Cloning	*Fam71f1*	F	ATGATGACATCAGTTCCACCTAGAAAGTC	
		R	TATAGAGTTTCCTCCAGTTAGGGACAGCC	
	Fam71f2	F	ATGAGTAAAATTAGGGGCCTCCCTCC	
		R	GGGTCCCAACAAGTTCTCTCC	
	Rab2a	F	ATGGCGTACGCCTATCTCTTCAAGTAC	
		R	TCAACAGCAGCCTCCCCCTGC	
	Rab2a (CA)	F	TTGGAGTCCTTTCGTTCTATCACACG	
		R	CCCTGCTGTATCCCAGATCTGG	
	Rab2a (CN)	F	AACTGCTTATTGCTACAGTTTACAG	
		R	TTTACCAACACCTGTGTCGC	
	Rab2b	F	ATGACTTACGCTTATCTCTTCAAGTACATCATCATC	
		R	GCAGCAGCCAGAGTCAGGCC	

Development • Supplementary information

Table S3. Sequences of gRNA.

Target	Name in Fig. 1	Sequence (5'→3')
Fam71f1 (Exon 2)	gRNA	CCTCCGTACCTTGCCTTCCC
Fam71f2 (5 'UTR)	gRNA_1	GCCCAACTCCAAGGTGTCGG
Fam71f2 (3' UTR - down stream region)	gRNA_2	AATGGAGTCCTCGTTGTTGG

Table S4. Antibodies used in this study.

Immunoprecipitation

Antigen	Provider	Catalog number	Volume
PA tag	FUJIFILM Wako Pure Chemical	016-25861	1 µL for Immunoblot sample<br>2 µL for Mass spectrometry sample
FLAG tag (M2)	Sigma-Aldrich	F1804	0.6 µL

Immunoblotting

Antigen	Provider or Reference	Catalog number	Dilution
PA tag	FUJIFILM Wako Pure Chemical	016-25861	1;1000
FLAG tag	MBL	PM020	1;1000
α-Tubulin (B-5-1-2)	Sigma-Aldrich	T5168	1;2000
β-Actin (AC-15)	abcam	ab6276	1;5000
GOPC	abcam	ab37036	1;1000
ZPBP1	Lin YN *et al*, Mol Cell Biol., 27:6794-805., (2007)		1;1000
SPACA1	Fujihara Y *et al*, Development, 139:3583-9., (2012)		1;1000
RAB2	Thermo fisher scientific	PA5-21962	1;500
Rabbit IgG (HRP conjugated)	Jackson ImmunoResearch	111-036-045	1;5000
Mouse IgG (HRP conjugated)	Jackson ImmunoResearch	115-036-062	1;5000
Rat IgG (HRP conjugated)	Jackson ImmunoResearch	112-035-167	1;5000
Goat IgG (HRP conjugated)	Jackson ImmunoResearch	805-035-180	1;500

Immunocytochemistry (ICC) and Immunohistochemistry (IHC)

Antigen	Provider or Reference	Catalog number	Dilution
HA tag	MBL	561	1;1000
TOM20 (FL-145)	Santa Cruz Biotechnology	sc-11415	1;100
IZUMO1 (KS64-125)	Ikawa M *et al*, J Biol Chem., 286:5639-46., (2011)		1;500 (Fig. 4C)<br>1;100 (Fig. 4D)
GM130	BD Biosciences	610822	1;1000
SPACA1	Fujihara Y *et al*, Development, 139:3583-9, (2012)		1;1000
RAB2	Thermo fisher scientific	PA5-21962	1;100
Rabbit IgG (Alexa Fluor 546 conjugated)	Thermo fisher scientific	A-11071	1;1000 (for ICC)<br>1;200 (for IHC)
Mouse IgG (Alexa Fluor 488 conjugated)	Thermo fisher scientific	A-11017	1;1000 (for ICC)<br>1;200 (for IHC)
Mouse IgG (Alexa Fluor 546 conjugated)	Thermo fisher scientific	A-11018	1;200
Rat IgG (Alexa Fluor 488 conjugated)	Thermo fisher scientific	A-11006	1;200
