# Supplementary material for: FAM71F1 binds to RAB2A and RAB2B and is essential for acrosome formation and male fertility in mice
Source: Development. 2021 Oct 29;148(21):dev199644. doi: 10.1242/dev.199644 (PMC8602946; doi:10.1242/dev.199644)
Supplement: Reviewer comments [file develop-148-199644_review_history.pdf]

FAM71F1 binds to RAB2A and RAB2B and is essential for acrosome formation and male fertility in mice

Akane Morohoshi, Haruhiko Miyata, Yuki Oyama, Seiya Oura, Taichi Noda and Masahito Ikawa

DOI: 10.1242/dev.199644

Editor: Haruhiko Koseki

Review timeline

Original submission:	6 May 2021
Editorial decision:	8 June 2021
First revision received:	31 August 2021
Accepted:	22 September 2021

Original submission

First decision letter

MS ID#: DEVELOP/2021/199644

MS TITLE: FAM71F1 binds to RAB2A and RAB2B and is essential for acrosome formation and male fertility in mice

AUTHORS: Akane Morohoshi, Haruhiko Miyata, Taichi Noda, and Masahito Ikawa

I have now received all the referees' reports on the above manuscript, and have reached a decision. The referees' comments are appended below, or you can access them online: please go to BenchPress and click on the 'Manuscripts with Decisions' queue in the Author Area.

As you will see, the referees express considerable interest in your work, but have some significant criticisms and recommend a substantial revision of your manuscript before we can consider publication. If you are able to revise the manuscript along the lines suggested, which may involve further experiments, I will be happy receive a revised version of the manuscript. Your revised paper will be re-reviewed by one or more of the original referees, and acceptance of your manuscript will depend on your addressing satisfactorily the reviewers' major concerns. Please also note that Development will normally permit only one round of major revision.

We are aware that you may be experiencing disruption to the normal running of your lab that make experimental revisions challenging. If it would be helpful, we encourage you to contact us to discuss your revision in greater detail. Please send us a point-by-point response indicating where you are able to address concerns raised (either experimentally or by changes to the text) and where you will not be able to do so within the normal timeframe of a revision. We will then provide further guidance. Please also note that we are happy to extend revision timeframes as necessary.

Please attend to all of the reviewers' comments and ensure that you clearly highlight all changes made in the revised manuscript. Please avoid using 'Tracked changes' in Word files as these are lost in PDF conversion. I should be grateful if you would also provide a point-by-point response detailing how you have dealt with the points raised by the reviewers in the 'Response to Reviewers' box. If you do not agree with any of their criticisms or suggestions please explain clearly why this is so.

© 2021. Published by The Company of Biologists under the terms of the Creative Commons Attribution License (https://creativecommons.org/licenses/by/4.0/).

Reviewer 1

Advance summary and potential significance to field

Although acrosome biogenesis during spermatogenesis has been well described the field lacks insight into molecular mechanisms of acrosome formation and function in vivo. In a well-written manuscript, Morohoshi et al., have focused on in vivo functions of Golgi-associated RAB2B interactor (GARI) and its binding protein GARI (encoded by Fam71f2) and GALI-L (encoded by Fam71f1) using gene-edited mice.

The Fam71f1 KO mice are infertile in vivo and although their sperm cannot penetrate the zona pellucida, ~30% can fuse with zona-free eggs in vitro.
Mature sperm had near normal motility but a grossly swollen acrosome with poorly localized IZUMO1 that is required for gamete fusion. The authors suggest that enhanced trafficking of acrosomal vesicles from the Golgi accounts for the swollen acrosome and document interaction of FAM71F1 with RAB2A/B in heterologous cells.

Comments for the author

1. Abnormalities of the Fam71f2 KO mice are incompletely described. Parallel experiments with the Fam71f2 KO should be undertaken and what is the phenotype of the double KO?

2. Although the tissue distribution and developmental expression of Fam71f1/2 transcripts are reported, there was no characterization of FAM71F1/2 proteins. If antibodies are not available, gene-edited mice could be used.

3. The Fig. 3 legend seems exaggerated and should be modified. As the authors describe in the text, the absence of Fam71f1 results in a malformed acrosome and defects in exocytosis but didn't dramatically affect IZUMO relocation.

4. Acrosome exocytosis in Fig. 3D needs confirmation by loss of the PNA signal or translocation of PSA to the equatorial segment in acrosome-reacted sperm. The IZUMO1 localization seems inconsistent in Fig. 3C and D.

5. Despite severe dysmorphology of mutant sperm (Fig. 4C and D), acrosome biogenesis seems comparable to controls (Fig. 4A and B) despite the description in the text. Although it is likely that Fam71f1 functions late in spermiogenesis where its abundance is greatest, the genesis of the swollen acrosome remains unclear and should be further investigated.

6. The molecular mass of SPACA1 and IZUMO1 changes during the acrosome reaction, and IZUMO1 translocates to the equatorial segment (Yamatoya et al., BOR, 2020). However, based on the rescue mice results, FAM71F1 is not present in mature sperm. How do the authors reconcile these observations with their conclusion that FAM71F1 is directly involved in SPACA1 cleavage and IZUMO1 translocation during the acrosome reaction?

7. Although the authors report that they have analyzed the proteome in mutant testis, there was no identification or characterization of candidate proteins causing the phenotype. Have the authors investigated RAB2B in mutant testes? What about heat-shock proteins?

Reviewer 2

Advance summary and potential significance to field

The manuscript entitled "FAM71F1 binds to RAB2A and RAB2B and is essential for acrosome formation and male fertility in mice" by Morohashi et al. show the role of FAM71F1 in the spermiogenesis, it has an essential role in formation of acrosome. In this study, the author showed that Fam71f1 KO mice show male infertility caused by disorders in acrosome formation, and that the infertility can be rescued by transgene of Fam71f1.

© 2021. Published by The Company of Biologists under the terms of the Creative Commons Attribution License (https://creativecommons.org/licenses/by/4.0/).

These results are very reliable and proves the importance of Fam71f1 in acrosome formation during spermiogenesis.
The molecular mechanisms during spermatogenesis, including acrosome formation, are still poorly understood, and this manuscript is an important contribution to the understanding of these mechanisms.

In this manuscript, however, the authors also tried to present data showing how Fam71f1 functions in acrosome formation, but it is unclear and insufficient. In addition, the manuscript shows that Fam71f1 binds to Rab2, but does not provide information on the signaling cascade of acrosome formation, such as whether Fam71f1 actually serves as a target molecule for Rab2 and leads to acrosome formation. These problems are not so serious as to undermine the importance of the study, but these should be revised before publication in Development.
Specific suggestions are given in "Suggestions to authors".

Comments for the author

Specific comments:
1. Page 6, lines 133-142 and Fig. 2C,D: Are the sperm head morphology abnormality in the KO mice found in all sperm, or is normal-shape sperm also observed? If all sperm have abnormal morphology, this should be clearly stated.
If some normal sperm are observed, the ratio of sperm with abnormal morphology should be clearly stated.
In addition, since morphology of the abnormal sperm looks similar, the two cases shown in Fig. 2C are acceptable, but since there seem to be many different patterns of acrosomal abnormalities, the number of cases shown in Fig.
2D should be increased.

2. Page 8, lines 184-186 and Fig. 3D: I'm not sure from the text here, but is it true that the disorder of relocation of IZUMO1 is seen in the KO sperm showing acrosome reaction? Further, was the acrosome of the sperm formed normally, or is the acrosome also abnormal as shown in Fig 2? This part needs to be rewritten so that it is easier to understand. If it is the former, it is worth showing this data, but if it is the latter, it is a natural result. Overall, this data is a superfluous experiment that deviates from the main subject of the manucript and I think it can be deleted, even though I understand importance of Izumo1 in fertilization.

3. Page 9, line 193 ?the abnormality appeared from around step10-11? and Fig. 4a: It is not clear what is "abnormality" and how it is "abnormality". The content of "abnormality" should be clearly described. Moreover, the difference between heterozygous and KO testes shown in Fig. 4A is not clear at a glance and cannot be understood, partly because there is no explanation in Fig. 4A. The figure should include arrows and other explanations so that readers in different specialties can clearly understand the differences. If possible, it would be better to replace Fig. 4A with data showing clear differences.

4. Page 9, line 198 "Abnormal swelling of the acrosome appeared around step" and Fig. 4B: It is not clear what is "Abnormal swelling acrosome? in Fig 4B. In my understand, Fig4B looks to be enlarged views of the Golgi apparatus.
Where is the the acrosome was being formed should be clearly indicated with arrows in Fig. 4B.
In addition, in Step 7-8, the Golgi apparatus of heterozygous spermatid appears to be more swollen. Is the photos of a heterozygous spermatid and a KO spermatid correct?

5. Page 9, line 199 "A swollen acrosome remained evident in the cauda epididymal spermatozoa" and Fig. 4C,D: It should be clearly indicated with arrows, etc. where the acrosome is in Fig. 4CD, although it is clearer than that in Fig. 4AB.

6. Page 9, lines 202-203 "These results suggest that membrane trafficking from the Golgi apparatus to the acrosome vesicle or the fusion of acrosomal granules is enhanced in the Fam71f1 mutants,": There is no evidence of "membrane trafficking from the Golgi apparatus" in the manuscript. It should be deleted.

© 2021. Published by The Company of Biologists under the terms of the Creative Commons Attribution License (https://creativecommons.org/licenses/by/4.0/).

7. Page 10, lines 215-228, and Fig. S6: It is said that there will be differences in the band pattern of SPACA1 between heterozygous and KO sperm, but it is not clear why SPACA1 forms multiple bands, thus an explanation should be added. In the discussion, there is a statement that the multi bands are due to glycosylation. If so, does digestion by glycosydases forms a single band? Is there any effect on the protein itself in KO mice?
Concerning Fig.S6B, I think sperm are also existed in the testis, but the pattern of sperm and testis is completely different especially in heterozygous mice. is SPACA1 a molecule processed in the epididymis? In addition, the band pattern of SPACA1 in heterozygous testis is expected to be the same as that of wildtype, but it is clearly different from the pattern of SPACA1 shown in Fujiwara et al. (2012).

8. In the manuscript, even though it was clearly shown that Fam71f1 binds only to the constitutive active form of Rab2, it is not clear that Fam71f1 actually acts as a target molecule for Rab2 during spermiogenesis. It is not a main subject in the manuscript, but if the author want to state the hypothesis that Fam71f1 acts in the acrosome formation induced by Rab2, add some more results that show the relationship more clearly.
Moreover, the signaling pathway for Rab-mediated membrane traffic is well established in many cells, so I recommend to add a more in-depth discussion. If Fam71f1 is involved in vesicular trafficking, does it localize to Golgi vesicles or acrosomal vesicles? Does Fam71f1 have a transmembrane domain? I understand that immunostaining of Fam71f1 is unable to perform due to lack of effective antibodies, but I would appreciate it if the authors show the information from the literatures and from its sequence.

Reviewer 3

Advance summary and potential significance to field

The authors found a new gene involved in acrosome biogenesis. This is a remarkable discovery due to the importance of this organelle during fertilization.

Comments for the author

This is a new paper from Ikawa's group where the authors investigate the role of a new gene that is important for the acrosome formation. They follow the same strategy that this group has been using: they focus on the testis-specific or enriched genes. Overall, this paper is excellent. They have created a transgenic animal to explore the role of this protein and found that is important for ccrosome biogenesis. Besides that, they have extensively analyzed the phenotype and all the results presented in this manuscript are consistent with its role in spermatogenesis. They have also explored the interaction of FAM71F1 with Rab2a/b.
I have carefully read the paper and I really do not have major comments (I. think this is the first time I do this).
The paper should be accepted as it is. I congratulate the author for such a good work.

First revision

Author response to reviewers' comments

We appreciate the constructive comments and suggestions the editor and reviewers have provided us. Because Yuki Oyama and Seiya Oura performed additional experiments such as *in vitro* fertilization, immunofluorescence, and immunoblotting, we added them as authors. Akane Morohoshi and Haruhiko Miyata are now equally contributed because Haruhiko Miyata also performed additional experiments and revised manuscript. All remaining authors agreed to this change.

We wrote responses to reviewers' comments below with the original comments in blue and our answers in black.

© 2021. Published by The Company of Biologists under the terms of the Creative Commons Attribution License (https://creativecommons.org/licenses/by/4.0/).

Reviewer 1 Advance Summary and Potential Significance to Field:
Although acrosome biogenesis during spermatogenesis has been well described, the field lacks insight into molecular mechanisms of acrosome formation and function in vivo. In a well-written manuscript, Morohoshi et al., have focused on in vivo functions of Golgi- associated RAB2B interactor (GARI) and its binding protein GARI (encoded by Fam71f2) and GALI-L (encoded by Fam71f1) using gene-edited mice.

The Fam71f1 KO mice are infertile in vivo and although their sperm cannot penetrate the zona pellucida, ~30% can fuse with zona-free eggs in vitro. Mature sperm had near normal motility but a grossly swollen acrosome with poorly localized IZUMO1 that is required for gamete fusion. The authors suggest that enhanced trafficking of acrosomal vesicles from the Golgi accounts for the swollen acrosome and document interaction of FAM71F1 with RAB2A/B in heterologous cells.

Thank you very much for your comments.

Reviewer 1 Comments for the Author:
1. Abnormalities of the Fam71f2 KO mice are incompletely described. Parallel experiments with the Fam71f2 KO should be undertaken and what is the phenotype of the double KO?

We analyzed IZUMO1 staining (fixed) (revised Fig. S3G), *in vitro* fertilization (revised Fig. 3B), sperm motility (revised Fig. S4C, D), and acrosome reaction (live IZUMO1 staining) (revised Fig. 3E, F) for *Fam71f2* KO mice. We did not check the phenotype of the double KO mice. Because it takes time to obtain double KO mice, we would like to leave it for a future study.

2. Although the tissue distribution and developmental expression of Fam71f1/2 transcripts are reported, there was no characterization of FAM71F1/2 proteins. If antibodies are not available, gene-edited mice could be used.

If antibodies are not available, we agree that it would be the best to generate knock-in mice that can be used for western blotting and immunofluorescence. However, because the transgenic approaches usually give high copy numbers and more protein amount, we chose to generate *Fam71f1-PA* Tg mice. Unfortunately, while the transgenically expressed FAM71F1-PA was physiologically functional, the protein could not be detected with anti- PA antibodies in immunofluorescence staining. Therefore, we need to examine which tag works best for immunofluorescence staining before generating KI mice. We would not like to include these challenging experiments in the present paper.

3. The Fig. 3 legend seems exaggerated and should be modified. As the authors describe in the text, the absence of Fam71f1 results in a malformed acrosome and defects in exocytosis but didn't dramatically affect IZUMO Relocation.

We changed the Fig. 3 legend to "*Fam71f1* but not *Fam71f2* is required for the acrosome reaction".

4. Acrosome exocytosis in Fig. 3D needs confirmation by loss of the PNA signal or translocation of PSA to the equatorial segment in acrosome-reacted sperm. The IZUMO1 localization seems inconsistent in Fig. 3C and D.

Because PNA signal is also disrupted in mutant spermatozoa (revised Fig. 2D), making it difficult to analyze the acrosome reaction, we performed live-sperm staining for IZUMO1 for both *Fam71f1* and *Fam71f2*. When we examined the IZUMO1 signal 15 minutes after incubating spermatozoa, no signals were observed in almost all the spermatozoa (revised Fig. 3C-F). In contrast, the IZUMO1 signal was observed 4 hours after incubating spermatozoa. These results indicate that IZUMO was exposed onto the plasma membrane during the acrosome reaction in both *Fam71f1* and *Fam71f2* mutant spermatozoa although the rate was lower in *Fam71f1* KO mice.

5. Despite severe dysmorphology of mutant sperm (Fig. 4C and D), acrosome biogenesis seems comparable to controls (Fig. 4A and B) despite the description in the text. Although it is likely that Fam71f1 functions late in spermiogenesis where its abundance is greatest, the genesis of the swollen acrosome remains unclear and should be further investigated.

© 2021. Published by The Company of Biologists under the terms of the Creative Commons Attribution License (https://creativecommons.org/licenses/by/4.0/).

We enlarged the pictures (revised Fig. 4A) and added arrowheads (revised Fig. 4A, B) to indicate abnormalities. Because there are no clear differences observed with immunofluorescence at step 2-5, the pictures were moved to revised Fig. S5A. Further, we showed additional pictures that indicate abnormally swollen acrosomes at step 4-5 with TEM (revised Fig. S5B).

6. The molecular mass of SPACA1 and IZUMO1 changes during the acrosome reaction, and IZUMO1 translocates to the equatorial segment (Yamatoya et al., BOR, 2020). However, based on the rescue mice results, FAM71F1 is not resent in mature sperm. How do the authors reconcile these observations with their conclusion that FAM71F1 is directly involved in SPACA1 cleavage and IZUMO1 translocation during the acrosome reaction?

We also think that FAM71F1 is not directly involved in SPACA1 cleavage and IZUMO1 translocation because FAM71F1-PA was not detected in the mature spermatozoa. To make this point clear, we added the sentences in the discussion section for SPACA1 (lines 292-296). For IZUMO1 translocation, we removed the sentences about abnormal IZUMO1 relocation because it is superfluous as Reviewer #2 mentioned.

7. Although the authors report that they have analyzed the proteome in mutant testis, there was no identification or characterization of candidate proteins causing the phenotype. Have the authors investigated RAB2B in mutant testes? What about heat-shock proteins?

Because it was difficult to choose specific heat shock proteins for further analyses, we focused on RAB2A/B. Using pan-RAB2 antibody that reacts both RAB2A and RAB2B (revised Fig. S8A), we confirmed that FAM71F1 interacts with RAB2A/B (revised Fig. 6A). Further, we found that the amount (revised Fig. 6B) or localization (revised Fig. 6C) of RAB2A/B in the testis was not impaired in *Fam71f1* KO mice.

Reviewer 2 Advance Summary and Potential Significance to Field:
The manuscript entitled "FAM71F1 binds to RAB2A and RAB2B and is essential for acrosome formation and male fertility in mice" by Morohashi et al. show the role of FAM71F1 in the spermiogenesis, it has an essential role in formation of acrosome. In this study, the author showed that Fam71f1 KO mice show male infertility caused by disorders in acrosome formation, and that the infertility can be rescued by transgene of Fam71f1. These results are very reliable and proves the importance of Fam71f1 in acrosome formation during spermiogenesis. The molecular mechanisms during spermatogenesis, including acrosome formation, are still poorly understood, and this manuscript is an important contribution to the understanding of these mechanisms.
In this manuscript, however, the authors also tried to present data showing how Fam71f1 functions in acrosome formation, but it is unclear and insufficient. In addition, the manuscript shows that Fam71f1 binds to Rab2, but does not provide information on the signaling cascade of acrosome formation, such as whether Fam71f1 actually serves as a target molecule for Rab2 and leads to acrosome formation. These problems are not so serious as to undermine the importance of the study, but these should be revised before publication in Development.
Specific suggestions are given in "Suggestions to authors".

Thank you very much for your comments. Using pan-RAB2 antibody that reacts with both RAB2A and RAB2B (revised Fig. S8A), we confirmed that FAM71F1 interacts with RAB2A/B (revised Fig. 6A). Further, we found that the amount (revised Fig. 6B) or localization (revised Fig. 6C) of RAB2A/B in the testis was not impaired in *Fam71f1* KO mice. Based on these experiments, we discussed the function of FAM71F1 and RAB2A/B in more detail (lines 302-313) as mentioned in the following response to comment #8.

1. Page 6, lines 133-142 and Fig. 2C,D: Are the sperm head morphology abnormality in the KO mice found in all sperm, or is normal-shape sperm also observed? If all sperm have abnormal morphology, this should be clearly stated. If some normal sperm are observed, the ratio of sperm with abnormal morphology should be clearly stated. In addition, since morphology of the abnormal sperm looks similar, the two cases shown in Fig. 2C are acceptable, but since there seem to be many different patterns of acrosomal abnormalities, the number of cases shown in Fig. 2D should be increased.

© 2021. Published by The Company of Biologists under the terms of the Creative Commons Attribution License (https://creativecommons.org/licenses/by/4.0/).

All spermatozoa exhibit abnormal head morphology in both *Fam71f1* and *Fam71f2* KO mice. We added sentences about the number of counted spermatozoa in the result section (lines 136-139). Further, we increased the number of sperm pictures for Fig. 2D (revised Fig. 2D, S3E).

2. Page 8, lines 184-186 and Fig. 3D: I'm not sure from the text here, but is it true that the disorder of relocation of IZUMO1 is seen in the KO sperm showing acrosome reaction? Further, was the acrosome of the sperm formed normally, or is the acrosome also abnormal as shown in Fig 2? This part needs to be rewritten so that it is easier to understand. If it is the former, it is worth showing this data, but if it is the latter, it is a natural result. Overall, this data is a superfluous experiment that deviates from the main subject of the manucript and I think it can be deleted, even though I understand importance of Izumo1 in fertilization.

We performed live-sperm staining for IZUMO1 for both *Fam71f1* and *Fam71f2*. When we examined the IZUMO1 signal 15 minutes after incubating spermatozoa, no signals were observed in almost all the spermatozoa (revised Fig. 3C-F). In contrast, the IZUMO1 signal was observed 4 hours after incubating spermatozoa. These results indicate that IZUMO1 was exposed onto the plasma membrane during the acrosome reaction in both *Fam71f1* and *Fam71f2* mutant spermatozoa although the rate was lower in *Fam71f1* KO mice. We agreed with the reviewer's comment and removed the sentences about abnormal IZUMO1 relocation.

3. Page 9, line 193 ?the abnormality appeared from around step10-11? and Fig. 4a: It is not clear what is "abnormality" and how it is "abnormality". The content of "abnormality" should be clearly described. Moreover, the difference between heterozygous and KO testes shown in Fig. 4A is not clear at a glance and cannot be understood, partly because there is no explanation in Fig. 4A. The figure should include arrows and other explanations so that readers in different specialties can clearly understand the differences. If possible, it would be better to replace Fig. 4A with data showing clear differences.

We enlarged the pictures and added arrowheads to indicate abnormally swollen acrosomes (revised Fig. 4A). Because there are no clear differences observed with immunofluorescence at step 2-5, the pictures were moved to revised Fig. S5A.

4. Page 9, line 198 "Abnormal swelling of the acrosome appeared around step" and Fig. 4B: It is not clear what is "Abnormal swelling acrosome? in Fig 4B. In my understand, Fig4B looks to be enlarged views of the Golgi apparatus. Where is the the acrosome was being formed should be clearly indicated with arrows in Fig. 4B. In addition, in Step 7-8, the Golgi apparatus of heterozygous spermatid appears to be more swollen. Is the photos of a heterozygous spermatid and a KO spermatid correct?

We added arrowheads to indicate abnormalities (revised Fig. 4B). Further, we showed additional pictures that indicate abnormally swollen acrosomes at step 4-5 with TEM (revised Fig. S5B). The photos of a heterozygous spermatid and a KO spermatid are correct.

5. Page 9, line 199 "A swollen acrosome remained evident in the cauda epididymal spermatozoa" and Fig. 4C,D: It should be clearly indicated with arrows, etc. where the acrosome is in Fig. 4CD, although it is clearer than that in Fig. 4AB.

We added arrowheads to indicate abnormalities (revised Fig. 4C, D).

6. Page 9, lines 202-203 "These results suggest that membrane trafficking from the Golgi apparatus to the acrosome vesicle or the fusion of acrosomal granules is enhanced in the Fam71f1 mutants,": There is no evidence of "membrane trafficking from the Golgi apparatus" in the manuscript. It should be deleted.

We removed this sentence.

7. Page 10, lines 215-228, and Fig. S6: It is said that there will be differences in the band pattern of SPACA1 between heterozygous and KO sperm, but it is not clear why SPACA1 forms multiple bands, thus an explanation should be added. In the discussion, there is a statement that the multi bands are due to glycosylation. If so, does digestion by glycosydases forms a single band? Is there

© 2021. Published by The Company of Biologists under the terms of the Creative Commons Attribution License (https://creativecommons.org/licenses/by/4.0/).

any effect on the protein itself in KO mice? Concerning Fig.S6B, I think sperm are also existed in the testis, but the pattern of sperm and testis is completely different, especially in heterozygous mice. is SPACA1 a molecule processed in the epididymis? In addition, the band pattern of SPACA1 in heterozygous testis is expected to be the same as that of wildtype, but it is clearly different from the pattern of SPACA1 shown in Fujiwara et al. (2012).

We tried PNGase F (revised Fig. S7C) and O-glycosidase (revised Fig. S7D) and found that N-linked glycosylation is abnormal in the *Fam71f1* mutant testis. After the PNGase F treatment, however, there were still several bands, which may be due to alternative splicing or other protein modifications. We added the sentences in the result section (lines 226-233). Further, SPACA1 was processed in the epididymis, which may be the secondary effect of abnormal acrosome formation. We added the sentences about this processing in the discussion section (lines 292-296, with track changes). The different band patterns are likely because SPACA1 signals were overexposed and overlapped in Fujihara et al. (2012).

8. In the manuscript, even though it was clearly shown that Fam71f1 binds only to the constitutive active form of Rab2, it is not clear that Fam71f1 actually acts as a target molecule for Rab2 during spermiogenesis. It is not a main subject in the manuscript, but if the author want to state the hypothesis that Fam71f1 acts in the acrosome formation induced by Rab2, add some more results that show the relationship more clearly. Moreover, the signaling pathway for Rab-mediated membrane traffic is well established in many cells, so I recommend to add a more in-depth discussion. If Fam71f1 is involved in vesicular trafficking, does it localize to Golgi vesicles or acrosomal vesicles? Does Fam71f1 have a transmembrane domain? I understand that immunostaining of Fam71f1 is unable to perform due to lack of effective antibodies, but I would appreciate it if the authors show the information from the literatures and from its sequence.

No transmembrane domain was found in FAM71F1, which was added in the result section (lines 245-246). Using the RAB2A/B antibody, we found that RAB2A/B was localized in the Golgi apparatus in the testis (revised Fig. 6C, S8B) when abnormal acrosome formation was observed with TEM (around step 4-5) (revised Fig. 4B). Considering that 1) FAM71F1 is localized in the Golgi apparatus in HEK293T cells (Fig. 5B) and 2) the acrosome is Golgi-derived organelle, the complex of active RAB2A/B and FAM71F1 in the Golgi apparatus may play roles in the acrosome formation. In *Drosophila,* it is suggested that active RAB2 is transported in Golgi-derived vesicles to fuse with RAB7- positive vesicles such as autophagosomes and late endosomes (PMID: 28483915). In moues testes, active RAB2A/B may also be transported in Golgi-derived vesicles to regulate their fusion with RAB7-positive acrosome vesicle. FAM71F1 may regulate this process negatively and its absence may enhance RAB2A/B-mediated acrosome formation. We discussed it in lines 302-313.

Reviewer 3 Advance Summary and Potential Significance to Field:
The authors found a new gene involved in acrosome biogenesis. This is a remarkable discovery due to the importance of this organelle during fertilization.

Reviewer 3 Comments for the Author:
This is a new paper from Ikawa's group where the authors investigate the role of a new gene that is important for the acrosome formation. They follow the same strategy that this group has been using: they focus on the testis-specific or enriched genes. Overall, this paper is excellent. They have created a transgenic animal to explore the role of this protein and found that is important for acrosome biogenesis. Besides that, they have extensively analyzed the phenotype and all the results presented in this manuscript are consistent with its role in spermatogenesis. They have also explored the interaction of FAM71F1 with Rab2a/b. I have carefully read the paper and I really do not have major comments (I. think this is the first time I do this).
The paper should be accepted as it is. I congratulate the author for such a good work.

Thank you very much for your comments.

© 2021. Published by The Company of Biologists under the terms of the Creative Commons Attribution License (https://creativecommons.org/licenses/by/4.0/).

Second decision letter

MS ID#: DEVELOP/2021/199644

MS TITLE: FAM71F1 binds to RAB2A and RAB2B and is essential for acrosome formation and male fertility in mice

AUTHORS: Akane Morohoshi, Haruhiko Miyata, Yuki Oyama, Seiya Oura, Taichi Noda, and Masahito Ikawa
ARTICLE TYPE: Research Article

I am happy to tell you that your manuscript has been accepted for publication in Development, pending our standard ethics checks.

Reviewer 1

Advance summary and potential significance to field

Although acrosome biogenesis during spermatogenesis has been well described the field lacks insight into molecular mechanisms of acrosome formation and function in vivo. In a well-written manuscript, Morohoshi et al., have focused on in vivo functions of Golgi-associated RAB2B interactor (GARI) and its binding protein GARI (encoded by Fam71f2) and GALI-L (encoded by Fam71f1) using gene-edited mice.

The Fam71f1 KO mice are infertile in vivo and although their sperm cannot penetrate the zona pellucida, ~30% can fuse with zona-free eggs in vitro.
Mature sperm had near normal motility but a grossly swollen acrosome with poorly localized IZUMO1 that is required for gamete fusion. The authors suggest that enhanced trafficking of acrosomal vesicles from the Golgi accounts for the swollen acrosome and document interaction of FAM71F1 with RAB2A/B in heterologous cells

Comments for the author

In the revised manuscript, the authors have addressed the concerns raised in the initial review.

Reviewer 2

Advance summary and potential significance to field

In the revised manuscript, it was clearly shown that acrosomal formation in the FAM71f1 KO mice have been disordered and that FAM71f1 interacts with RAB2.
The revision greatly increases the reliability of this study and proves the importance of FAM71f1 in the acrosome formation during spermiogenesis.

Comments for the author

In the revised manuscript, the indicated points by the reviewers have been carefully revised with additional experiments, and the insufficient points have been almost completely resolved.
I am fully satisfied with the revised version, and think that it may be accepted in the present form.

Reviewer 3

Advance summary and potential significance to field

N/A

Comments for the author

The paper should be accepted.

© 2021. Published by The Company of Biologists under the terms of the Creative Commons Attribution License (https://creativecommons.org/licenses/by/4.0/).
